# Optimized image preprocessing strategies for enhanced neural network-based defect detection in industrial automation

**DOI:** 10.1038/s41598-026-50951-y

**Published:** 2026-07-06

**Authors:** Sai Prakash Challa, Melvin Alexis Lara de Leon, Jiri Koziorek, Ibrahim A. Hameed, Zdenek Machacek

**Affiliations:** 1 Department of Cybernetics and Biomedical Engineering, Faculty of Electrical Engineering, Computer Science and Cybernetics, VSB-Technical University of Ostrava, 17. Listopadu 2172/15, 70800 Ostrava, Czechia; 2 Department of Mechanical Engineering and Technology Management, Faculty of Science and Technology, Norwegian University of Life Sciences (NMBU), Drøbakveien 31, 1433 Ås, Norway

**Keywords:** Machine vision, Defect detection, Genetic algorithm, Deep learning, Industrial quality control, Manufacturing automation, Production efficiency, Engineering, Mathematics and computing

## Abstract

Machine vision and AI-based defect detection systems are increasingly deployed in manufacturing to support consistent product quality and high production efficiency. However, these automated inspection systems often suffer from sensitivity to imaging variability, dependence on large labeled datasets, and the need for manually engineered preprocessing pipelines–limitations that hinder accuracy and reliability in real industrial conditions. This study presents a novel approach for optimizing image preprocessing strategies for neural network–based defect detection using a genetic algorithm (GA)-driven evolutionary framework. The method systematically explores a set of 48 preprocessing operations and automatically evolves optimal filter sequences through multi-objective fitness evaluations incorporating classification accuracy, computational efficiency, and preprocessing robustness. The genetic algorithm generates diverse preprocessing sequences of varying lengths (3–6 filters) and evaluates a broad range of population sizes (20–100 individuals) and generation limits (20–150 generations) to identify configurations that maximize detection performance while reducing data requirements. Extensive experiments across three product categories show that GA-optimized preprocessing significantly outperforms raw-image baselines and manually designed preprocessing pipelines. Results demonstrate substantial gains in classification accuracy (up to 15%) and improved data efficiency, requiring 30–60% fewer training images to achieve target performance. The findings confirm that evolutionary optimization provides a robust and scalable solution for industrial defect detection, enabling more reliable and efficient machine vision systems for modern manufacturing environments.

## Introduction

Machine vision integrated with artificial intelligence (AI)-based defect detection is widely utilized in manufacturing and production environments to ensure product quality, regulatory compliance, and operational efficiency. These automated inspection systems play an instrumental key role in detecting, identifying, and localizing defects, thus maintaining consistency and reliability throughout the production cycle. As demand for precision and automation continues to grow, improving the accuracy and robustness of defect detection systems is essential to reduce variability in final products and maximize efficiency through early preventive and corrective measures to minimize defective products^1,2^.

However, a critical challenge in automated inspection lies in optimizing the accuracy and reliability of neural network-based defect detection systems, as their performance is inherently dependent on the quality and quantity of input/training data. The selection of appropriate preprocessing techniques requires domain knowledge of each product and the nature of defects to be detected, where manual selection approaches are both time-consuming and cumbersome. Recent advances in evolutionary computation have demonstrated significant potential in addressing these challenges through automated optimization of preprocessing pipelines.

To address this challenge, this study aims to improve the quality of the input data through a comprehensive evaluation of three distinct methodologies: raw image training, manual selection of preprocessing techniques, and genetic algorithm-based optimization. The objective is to advance the field of machine vision by developing and evaluating innovative preprocessing strategies that improve the performance of neural networks. This study focuses on two critical tasks: defective (D) vs. non-defective (ND) quality detection, and defect localization, which are essential for maintaining product quality, enabling efficient automated inspection, and meeting the growing demands for accuracy and reliability in industrial applications.

**Fig. 1:**
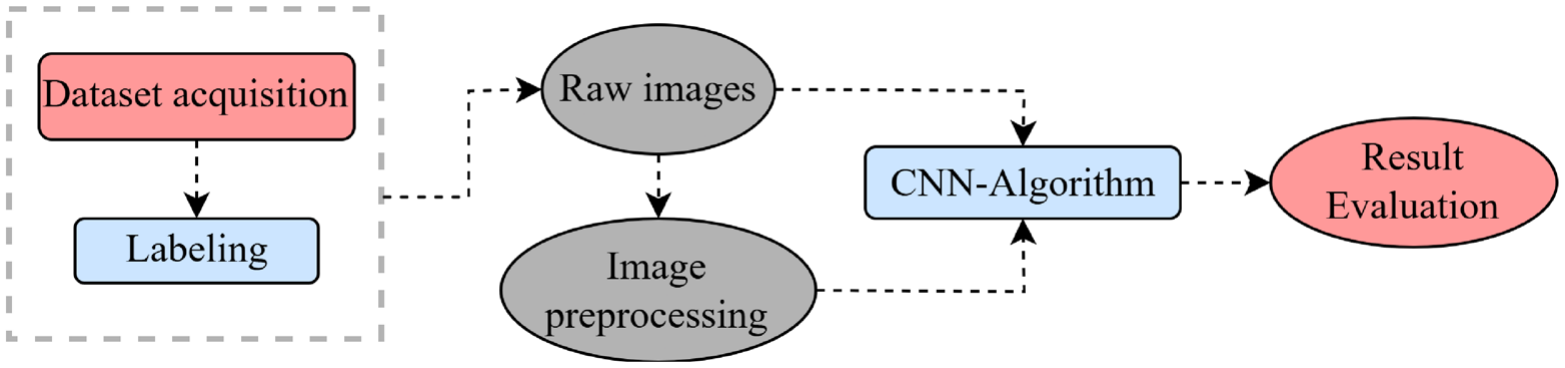
Conceptual framework.

Figure 1 illustrates the development pipeline which begins with data acquisition, where images of each product type are collected to ensure that the dataset reflects various scenarios, defect types, environmental conditions and product variations commonly encountered in real-world applications. These images are then systematically labeled and preprocessed to prepare them for model training, testing, and validation using three distinct approaches.

The first methodology establishes a baseline by training convolutional neural network (CNN) models directly on raw, unprocessed images without any preprocessing interventions. This approach provides a fundamental performance benchmark against which the effectiveness of subsequent preprocessing strategies can be evaluated.

The second methodology employs manual selection of preprocessing techniques, where domain experts systematically choose and configure preprocessing filters based on their knowledge of specific product characteristics and defect types. This traditional approach involves techniques such as edge detection, noise reduction, morphological operations, and contrast enhancement, tailored to the unique characteristics of each product category to enrich images with relevant features for subsequent analysis and training^3,4^.

The third methodology introduces a novel genetic algorithm-based optimization framework that automatically evolves optimal filter preprocessing sequences without requiring extensive manual intervention. The genetic algorithm systematically explores various combinations of preprocessing parameters and filter configurations through evolutionary computation principles, including selection, crossover, and mutation operations^5–7^. This approach generates diverse preprocessing sequences of varying lengths (typically 3-6 filters), evaluating their effectiveness through fitness functions that assess both detection accuracy and computational efficiency. The genetic algorithm maintains a population of preprocessing pipelines, iteratively improving them through successive generations until the convergence criteria are met^8,9^.

The effectiveness of the proposed methodologies is evaluated through a comprehensive comparative study in which CNN models are trained using images processed by each of the three approaches. Performance is compared across multiple preprocessing strategies, starting with the raw image baseline, progressing through manually selected preprocessing pipelines, and culminating with genetically optimized sequences. Additionally, the study evaluates the impact of the size of the training dataset on the performance of the model, analyzing how variations in the number of training images per class influence accuracy and efficiency between different preprocessing methodologies^10–12^.

To achieve systematic assessment, a stratified 80/20 training-to-test split is applied, where 80% of images are used for training and 20% are reserved for testing. The number of training images is systematically varied by randomly selecting n images per product class, where n ranges from minimal training sets to the full available training data, while maintaining the established train-to-test ratio. This approach enables the identification of the optimal number of training images required for superior model performance under each preprocessing strategy.

In the final phase, performance metrics from all three methodologies are compared using standard evaluation criteria, including accuracy, precision, recall, and F1-score. The research identifies the most effective preprocessing approach and determines the optimal size of the training dataset required to achieve superior performance for each category of products. This evaluation highlights the influence of automated optimization versus manual selection on model accuracy, efficiency, and reliability.

In general, this study highlights the critical role of optimization methodologies in improving neural network performance for industrial defect detection tasks. The findings contribute to the advancement of automated quality inspection processes by demonstrating how genetic algorithm-based preprocessing optimization can reduce training complexity while improving accuracy and computational efficiency compared to traditional manual approaches. By addressing challenges such as dataset variability and the need for robust automation solutions, this research establishes a foundation for advancing machine vision technologies in diverse industrial applications, particularly in scenarios where rapid adaptation to new types of defects and production environments is essential.

## Related work

Traditional image preprocessing methods for industrial defect detection have relied heavily on manual parameter selection and domain expertise. Classical approaches employ well-established techniques including morphological operations (erosion, dilation, opening, closing), edge detection algorithms (Sobel, Canny, Roberts operators), geometric transformations, and noise reduction filters. These methods have demonstrated effectiveness in specific scenarios, with morphological operations achieving accuracies of 88.2% in wood defect detection^13^, although precision remained limited at 62.8%. Edge detection using Canny operators achieved a higher precision of 84.7%, but suffered from reduced recall.

However, traditional approaches face significant limitations. They require extensive manual feature engineering and parameter tuning, struggle with complex or varying defect patterns, and exhibit sensitivity to environmental conditions such as lighting variations. The preprocessing pipeline for the detection of steel plate surface defects exemplifies these challenges^14^, requiring manual threshold setting and preset parameter configuration for the detection of the region-of-interest. Furthermore, traditional methods such as improved adaptive median filtering require boundary filling, grayscale judgment, and manual filter window size adjustment, which makes them computationally intensive and difficult to adapt to new defect types^15^.

### Deep learning for industrial defect detection

Convolutional Neural Networks have emerged as the dominant approach for industrial defect detection due to their hierarchical feature learning capabilities and spatial pattern recognition. Recent advances demonstrate that CNN-based systems can achieve exceptional performance when combined with appropriate preprocessing strategies. Transfer learning has proven particularly effective in industrial applications, allowing rapid adaptation to new product lines with minimal training data requirements^16,17^.

Deep transfer learning approaches have shown remarkable success in various industrial domains. Research has demonstrated that R-CNN models combined with AlexNet achieve superior defect detection accuracy^18^, particularly when enhanced with transfer learning methodologies. Transfer learning enables models to adapt from source domains to target applications, requiring only limited new samples instead of extensive retraining. Studies have shown that transfer learning improves welding defect detection accuracy by using transferred weights, which increases the variance and contrast of the feature map.

Despite these advances, CNN-based approaches face critical challenges. They require substantial computational resources and large labeled datasets for optimal performance. More importantly, their effectiveness is inherently dependent on the quality of input preprocessing, creating a bottleneck where the selection of manual preprocessing parameters becomes a limiting factor. This dependency highlights the need for automated preprocessing optimization to fully exploit CNN capabilities.

### Automated preprocessing optimization

Recent research has increasingly focused on automated preprocessing optimization to address the limitations of manual parameter selection. Automated machine learning pipelines have emerged as powerful tools for streamlining data preprocessing, feature engineering, and model optimization. However, most existing automated preprocessing frameworks focus on structured data rather than image preprocessing for defect detection applications.

#### Meta-heuristic optimization methods

Meta-heuristic algorithms have demonstrated significant potential in addressing complex optimization problems across various domains. These algorithms provide flexible and adaptive approaches with extensive search capabilities, making them suitable for NP-hard problems, including image processing optimization. Recent comprehensive reviews have identified over 540 meta-heuristic algorithms, demonstrating the rapid evolution of this field. Applications span fixture design, cell manufacturing optimization, robotics, and medical science, indicating their versatility^19^.

Chaotic Crow and Krill Herd optimization has been successfully applied to enhance image contrast, demonstrating superior performance in terms of contrast, edge details, and structural similarity compared to traditional methods. However, most meta-heuristic applications in image processing focus on specific aspects such as contrast enhancement or segmentation rather than comprehensive preprocessing pipeline optimization^20^.

The design of an effective image preprocessing pipeline requires making discrete filter selections, tuning continuous parameters, and determining the appropriate number and ordering of operations. A Genetic Algorithm (GA) was selected to optimize this pipeline because the task involves discrete operator choices, continuous parameter tuning, and variable-length sequences–characteristics that together create a highly irregular search space. Such mixed and dynamic structures are difficult to handle with methods like Bayesian Optimization, which typically assumes a fixed-dimensional continuous domain and therefore struggles with variable-length pipelines^21^. Particle Swarm Optimization faces similar limitations: although effective for numerical optimization, adapting it to discrete and ordered operations requires substantial modification that often reduces overall efficiency^22^. Existing AutoML frameworks primarily target Neural Architecture Search (NAS), and dedicated tools for sequential image preprocessing remain limited^23^. In contrast, the GA framework naturally supports mixed-variable representations and flexible encodings, allowing joint optimization of pipeline composition, operator ordering, and parameter settings. Its ability to accommodate variable-length structures through intuitive crossover and mutation makes GA particularly well-suited for exploring complex combinatorial pipelines, where the effectiveness of each preprocessing step depends strongly on both its configuration and its position within the sequence.

#### Genetic algorithm applications in image processing

Genetic algorithms have shown particular promise in image processing optimization tasks. Recent research has demonstrated successful applications in image segmentation, where genetic algorithms optimize hyperparameters of Gabor filters combined with random forest classifiers, achieving superior F1-scores compared to alternative methods. The key advantage lies in treating hyperparameters as genes within chromosomes, allowing evolutionary optimization of filter characteristics^24,25^.

Genetic algorithms have also proven to be effective in feature selection for image preprocessing, with improved PCA combined with genetic algorithms solving time-consuming problems in traditional approaches. Variable length genetic algorithms have been particularly successful in CNN hyperparameter optimization^26^, systematically tuning parameters to improve performance while handling variable-depth architectures. However, existing genetic algorithm applications typically focus on single aspects of preprocessing rather than comprehensive pipeline optimization^5^.

#### Evolutionary CNN optimization

The integration of evolutionary algorithms with CNN optimization has gained significant attention for hyperparameter tuning and architecture optimization. Evolutionary approaches address the discrete, non-continuous nature of hyperparameter spaces where traditional gradient-based methods fail. Research has demonstrated that genetic algorithms can effectively optimize CNN configurations, including convolutional layer parameters, fully connected layer sizes, learning rates, and regularization parameters^27,28^.

Enhanced Battle Royale Optimization (EBRO) algorithms have been successfully integrated with CNNs, employing proportional derivative (PD) controllers to improve optimization efficiency^29^. Variable length genetic algorithms have proven particularly effective for CNN hyperparameter optimization, handling variable numbers of parameters as model depth increases. Multi-objective genetic algorithms have also been developed to optimize both accuracy and computational efficiency simultaneously^30^.

However, existing evolutionary CNN optimization focuses primarily on network architecture and training hyperparameters rather than optimization of the preprocessing pipeline. This represents a significant gap where preprocessing remains manually configured despite the success of evolutionary approaches in other aspects of CNN optimization.

### Hybrid approaches and recent advances

Recent research has increasingly explored hybrid approaches that combine multiple optimization strategies for improved performance. Hybrid genetic algorithms integrated with deep learning techniques have demonstrated superior performance in various applications, efficiently navigating complex hyperparameter search spaces. Gray Wolf Optimizer (GWO), Particle Swarm Optimization (PSO), and Genetic Algorithms (GA) have been successfully combined with CNN architectures for simultaneous preprocessing and network optimization^31,32^.

Multi-objective optimization approaches have emerged as particularly promising, addressing the trade-off between detection accuracy and computational efficiency. Genetic algorithm optimization of ensemble learning methods has shown improved classification accuracy while maintaining computational feasibility. However, most hybrid approaches focus on model optimization rather than comprehensive pipeline preprocessing automation^33,34^.

### Research gaps and motivation

Although AI-driven machine vision has advanced considerably in recent years, key limitations in preprocessing design, optimization, and practical deployment continue to restrict the development of reliable, high-performance defect detection systems.

- *Limited Integration of Preprocessing Optimization:* While genetic algorithms have proven successful in CNN hyperparameter optimization and image processing tasks, there is insufficient research on comprehensive preprocessing pipeline optimization specifically for industrial defect detection. Most approaches focus on either preprocessing techniques or CNN optimization independently, missing the synergistic benefits of integrated optimization.
- *Manual Parameter Selection Persistence:* Traditional defect detection systems continue to rely on manually crafted preprocessing pipelines with expert-selected parameters. This limitation persists despite the availability of sophisticated optimization algorithms, creating a bottleneck in achieving optimal detection performance.
- *Lack of Systematic Comparison Frameworks:* Existing research lacks comprehensive frameworks that systematically compare raw image training, manual preprocessing optimization, and automated evolutionary approaches within the same experimental context. This gap makes it difficult to quantify the benefits of automated preprocessing optimization.
- *Industrial Applicability Gap:* While meta-heuristic optimization shows promise in theoretical applications, there is limited research on practical implementation in industrial defect detection scenarios with real-world constraints. Most studies focus on benchmark datasets rather than industrial manufacturing environments.

These gaps motivate our research approach, which introduces a comprehensive genetic algorithm-based framework for automated preprocessing pipeline optimization. Our methodology addresses the limitation of manual parameter selection by automatically evolving optimal preprocessing sequences, while providing a systematic comparison against traditional approaches. The integration of GA optimization with CNN-based defect detection represents a novel contribution that bridges the gap between evolutionary computation advances and practical industrial applications.

## Methodology

This section outlines the comprehensive experimental framework employed to develop and evaluate optimized image preprocessing strategies for neural network-based defect detection in industrial automation. The approach integrates systematic data acquisition, distinct preprocessing paradigms, genetic algorithm optimization, convolutional neural network architecture, and rigorous statistical evaluation methodologies^35,36^.

The experimental design follows established principles for computer vision research, incorporating stratified sampling, controlled variables, and statistical frameworks for model evaluation. Our methodology adopts a design science approach that operates at the interface of creative design and explanatory science to create and test innovative preprocessing optimization solutions. The experimental framework consists of stratification, sampling, and estimation components that allow efficient model evaluation while maintaining statistical rigor.

### Dataset acquisition and partitioning

In data acquisition, images of all required products are captured using a Basler sensor and a Baumer camera under varying lighting conditions and stored according to the respective product categories. For the use case presented in this article, the dataset is organized into three product categories, each divided into two classes: Defective (D) and Non-Defective (ND), as shown in Table 1.

Dataset partitioning follows stratified sampling methodologies to ensure proportional representation across all stratification criteria while maintaining the statistical rigor necessary for unbiased model training and evaluation. The experimental dataset comprises 543 images (297 defective, 246 non-defective) distributed across three industrial products: Product 1 (111 defective, 94 non-defective), Product 2 (65 defective, 80 non-defective), and Product 3 (121 defective, 72 non-defective). For Product 1, the defective class includes multiple visually distinct defect types (e.g., surface scratches, local contamination, and shape irregularities), whereas Product 2 exhibits fewer but more homogeneous defect patterns. In contrast, Product 3 primarily contains a single dominant defect type with relatively consistent appearance across samples. Lighting conditions also differ across products: images for Products 1 and 2 were captured in a laboratory setup with fixed but moderately controlled illumination, while Product 3 was acquired directly on an industrial production line using a dedicated machine-vision lighting arrangement with highly uniform illumination. Each product therefore exhibits a moderate but realistic class imbalance, and stratified partitioning was applied to preserve the original defective/non-defective ratios in both training and test subsets. A stratified 80/20 training-to-test split was consistently applied in all experimental settings, resulting in approximately 434 training images and 109 test images throughout the full dataset.

To evaluate how the size of the training set influences model performance, the number of training images was systematically varied by randomly selecting *n* images per product class, where *n* corresponds to predetermined sample sizes (8, 12, 16, 20, 24, 28, and 32 images for different products) while maintaining the stratified split ratio throughout each experiment. This approach enables the identification of the minimum effective sample size required for each preprocessing condition and product category.

**Table 1:**
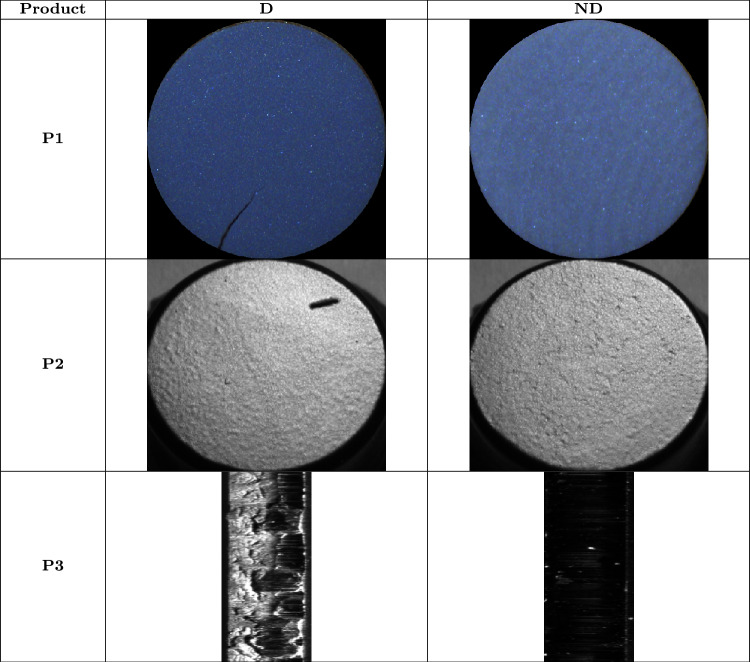
Sample images of products with class labels defective (D) and non-defective (ND).

### Image preprocessing methodologies

The investigation employs three distinct preprocessing paradigms that enable a comprehensive comparative analysis of automated versus manual optimization approaches, providing empirical evidence for the effectiveness of the evolutionary algorithm in industrial computer vision applications. As shown in Table 1, each product can be classified into two classes–Defective (D) and Non-Defective (ND)–with the images serving as representative samples for each class.

#### Baseline methodology: raw image processing

**Fig. 2:**
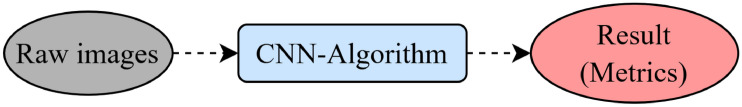
Raw image processing.

As illustrated in Fig. 2, the baseline condition uses unprocessed images as direct CNN input, establishing fundamental performance benchmarks against which preprocessing interventions are evaluated. Raw image processing maintains original pixel intensities, spatial dimensions, and color characteristics as acquired from imaging systems, ensuring that baseline results do not suffer artificial enhancement bias. Essential normalization operations include scaling the value of the pixels in a range and standardized resizing to uniform dimensions using bilinear interpolation while preserving aspect ratios.

#### Manual preprocessing optimization

**Fig. 3:**
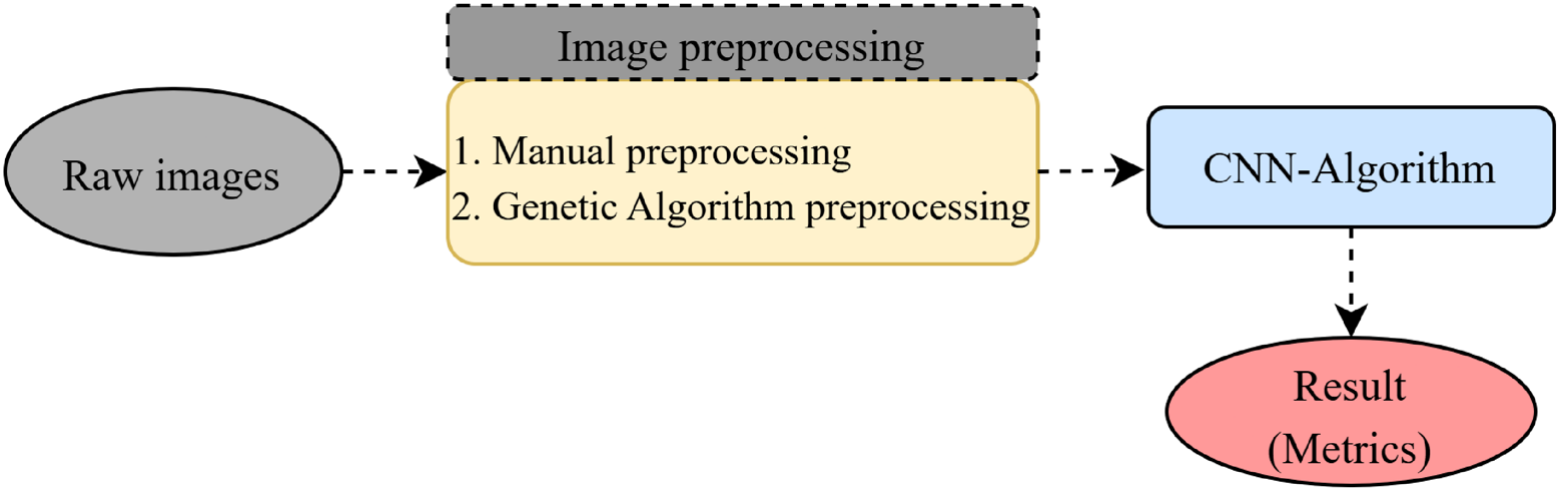
Workflow for image preprocessing.

As illustrated in Fig. 3, traditional preprocessing is based on systematic expert-driven parameter selection informed by domain knowledge and established industrial computer vision practices. The manual optimization process involves designing comprehensive preprocessing pipelines, where computer vision specialists iteratively evaluate and refine filter combinations through trial-and-error to identify the most effective configuration for each product category.

*Comprehensive Filter Library* Manual preprocessing utilizes a comprehensive library of 48 distinct preprocessing operations shown in Table 2, including binarization with threshold optimization, morphological operations (dilation, erosion, opening, closing), edge detection methods (Sobel, Canny, Roberts, Prewitt, DoG, Laplacian), filtering techniques (Gaussian, median, bilateral, low-pass, high-pass), geometric transformations (scaling, rotation, translation), and advanced techniques (watershed segmentation, wavelet transforms, active contours).

*Parameter Optimization Process* Each preprocessing operation requires careful parameter tuning based on the characteristics of each product category. For example, binarization thresholds are selected using histogram-based intensity distributions (typical ranges: 80–140). Morphological operations employ kernel sizes between 3$\times$3 and 11$\times$11 pixels, chosen according to the shape and scale of the defect, while the edge detection parameters are adjusted based on local edge-strength statistics.

**Table 2:** Comprehensive set of image preprocessing filters and transformations used in this study.

Preprocessing methods		
0. Binarization	16.Average Filter	32. Watershed
1. Conversion Character	17.Lowpass Filter	33. Closing Operation
2. Equalized Image	18. Recursive Filter	34.Dilation
3. Enhanced Image	19. Separable Filter	35. Erosion
4. Integral Fourier	20. Upperpass Filter	36. Opening Operation
5. Normalization	21. Affine Transform	37. Histogram Equalization
6. Noise Smoothing	22. Bilinear Interpolation	38.Nearest Neighbour
7. DoG Edge Detector	23. Image Shifting	39. Mode Filter
8. Laplacian	24. Nearest Neighbor Interpolation	40. Median Filter
9. Marr-Hildreth Edge Detector	25. Rotation	41. Bilateral Filter
10. Canny Edge Detector	26. Scaling	42. Gaussian Filter
11. Deriche Edge Detection	27. Skeletonization	43. WatershedBG
12. Krisch Edge Detection	28. WaveletAPP	44. Watershedunknown
13. Prewitt Edge Detection	29. Active Contours	45. Wavelet-HD
14. Roberts Edge Detection	30. Edge Detection Methods	46. Wavelet-VD
15. Sobel Edge Detection	31. Parametric Threshold	47. Wavelet-APP-DD

*Sequence design methodology* Manual preprocessing pipelines are designed for each product category using domain expertise and typically consist of **3–7 sequential operations**. These sequences are tailored to the visual characteristics of each product and the expected defect patterns. For example, preprocessing for *Product 1* may involve combinations such as [binarization $\rightarrow$ enhanced image $\rightarrow$ dilation $\rightarrow$ erosion $\rightarrow$ opening], each with parameter settings optimized for that product’s defect morphology.

**Fig. 4:**
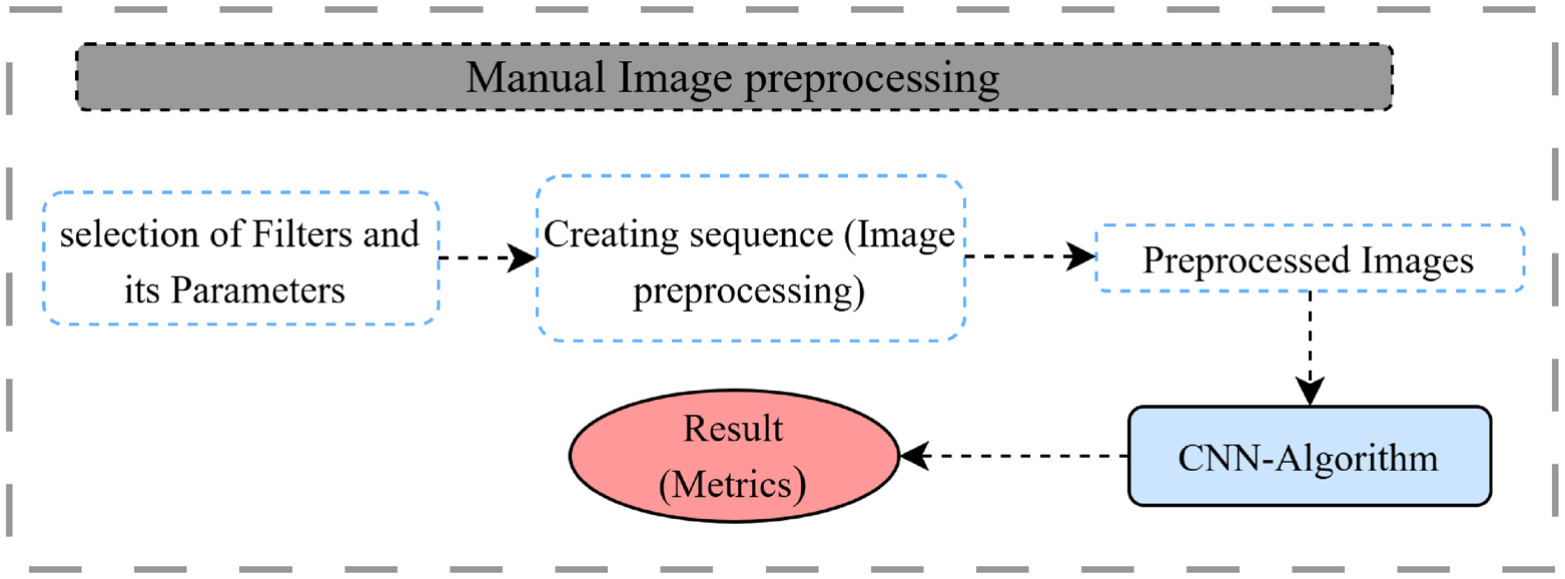
Workflow for manual image preprocessing, showing the sequential design and transformation of input images.

To illustrate the input format, consider a sequence of five filters *: binarization, erosion, dilation, skeletonization, and low-pass filtering*. This sequence is encoded as:

$$[0,\ 33,\ 34,\ 28,\ 17],$$

where each value corresponds to a specific operation in the preprocessing library. The output of each filter automatically becomes the input of the next, producing a step-wise transformation pipeline. This manual design workflow, including operation selection, ordering, and parameter tuning, is illustrated in Fig. 4.

*Advantages of manual selection* Manual selection provides a structured and intuitive approach for designing preprocessing pipelines, particularly when expert insight is essential.

- Offers direct control over the choice and application of preprocessing techniques.
- Allows for customization tailored to the unique characteristics of the product.
- Ideal for applications with predictable and consistent image properties.

*Limitations of Manual Selection* Despite its usefulness, manual selection introduces several practical and methodological drawbacks that can impact efficiency and consistency.

- Demands a high level of expertise and experience.
- Can be time-consuming, as it requires testing various filter combinations manually.
- Susceptible to inconsistencies due to human subjectivity and judgment.

*Product 1 Manual Preprocessing* For Product 1, a tailored preprocessing sequence was developed to address its specific visual properties and defect characteristics. After several iterations, the following sequence was selected: *Sequence (17, 2, 3, 6, 0)*

Each index in the sequence corresponds to a distinct operation in the preprocessing library, as detailed below:

- **Low-Pass Filter** (17) with a cutoff frequency of **80**
- **Equalized Image** (2)
- **Enhanced Image** (3) with **sigma = 1**
- **Noise Smoothing** (6) with:
  - **Kernel Size**: $$9 \times 9$$
  - **Sigma**: 1
- **Binarization** (0) with a threshold value of **80**

**Fig. 5:**
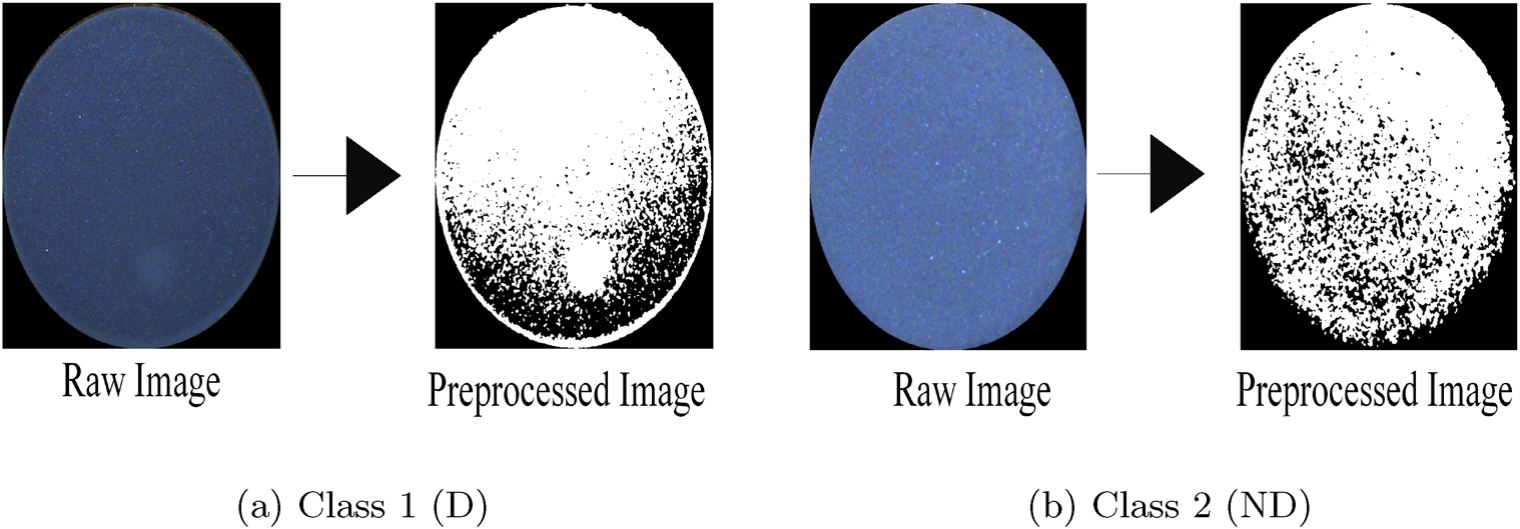
Images of Defective (D) and non-defective (ND) of product 1 before and after preprocessing method (17, 2, 3, 6, 0): (**a**) Class 1 (D); (**b**) Class 2 (ND).

The results generated by this preprocessing pipeline are presented in Fig. 5, which illustrates its effectiveness in improving the images of the product for subsequent classification.

***Product 2 Manual Preprocessing***

*Sequence: (17, 0, 34)*

The preprocessing sequence applied for Product 2 is **(17, 0, 34)**, which corresponds to the following filters:

- **Low-Pass Filter** (17) with a cutoff frequency of **140**
- **Binarization** (0) with a threshold value of **100**
- **Dilation** (34) using a kernel size of $3 \times 3$

**Fig. 6:**
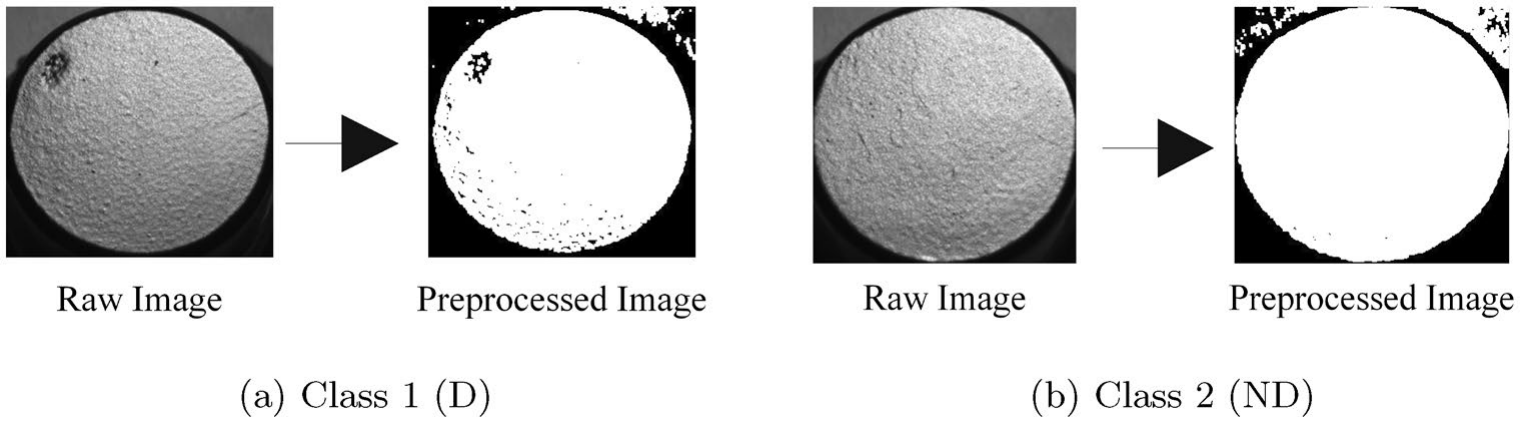
Images of Defective (D) and non-defective (ND) of product 2 before and after preprocessing method (17, 0, 34): (**a**) Class 1 (D); (**b**) Class 2 (ND).

The visual output of this sequence for classes 1 and 2 is shown in Fig. 6.

***Product 3 Manual Preprocessing***

*Sequence: (17, 14, 0, 3)*

The preprocessing sequence applied for Product 3 is **(17, 14, 0, 3)**, corresponding to the following filters and parameters:

- **Low-Pass Filter** (17) with a cutoff frequency of **140**
- **Robert’s Edge Detection** (14)
- **Binarization** (0) with a threshold value of **30**
- **Enhanced Image** (3)

**Fig. 7:**
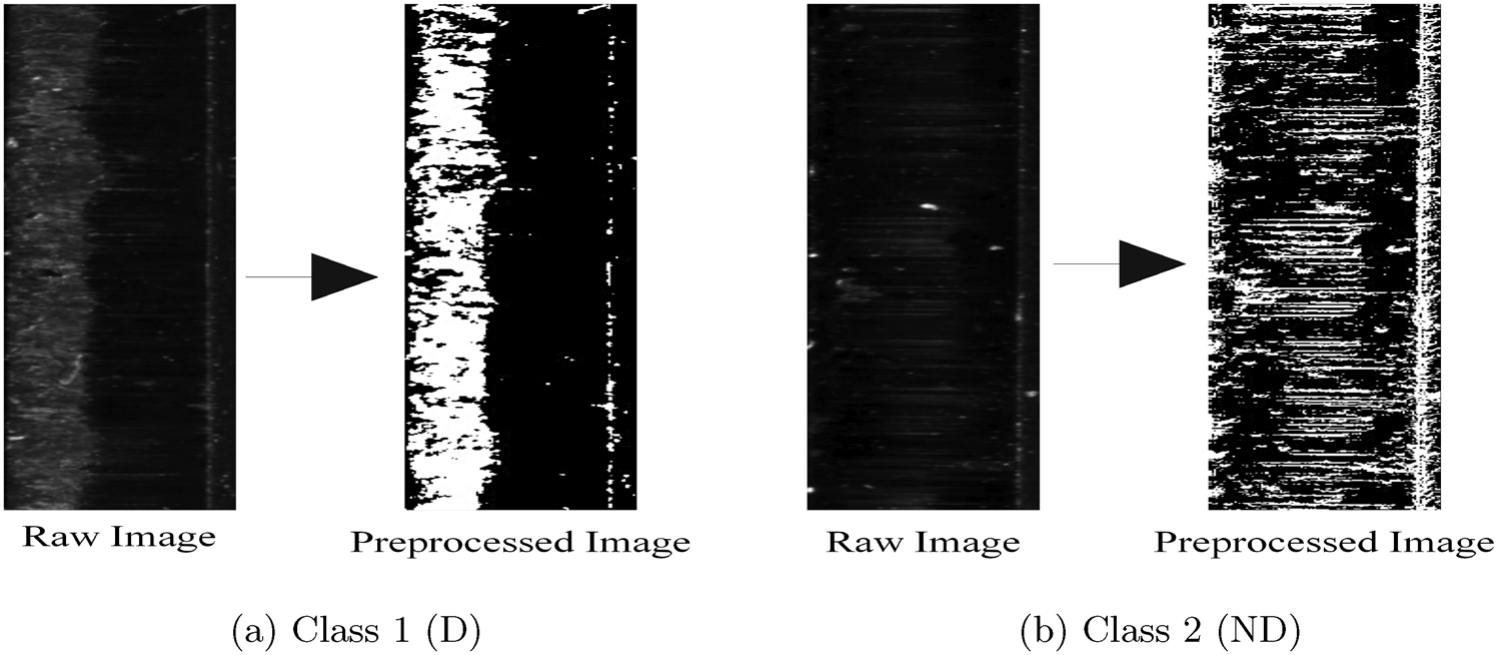
Images of Defective (D) and non-defective (ND) of product 3 before and after preprocessing method (17, 14, 0, 3): (**a**) Class 1 (D); (**b**) Class 2 (ND).

Figure 7 presents the visual outputs of this sequence applied to all three classes.

#### Genetic algorithm-based preprocessing optimization

**Fig. 8:**
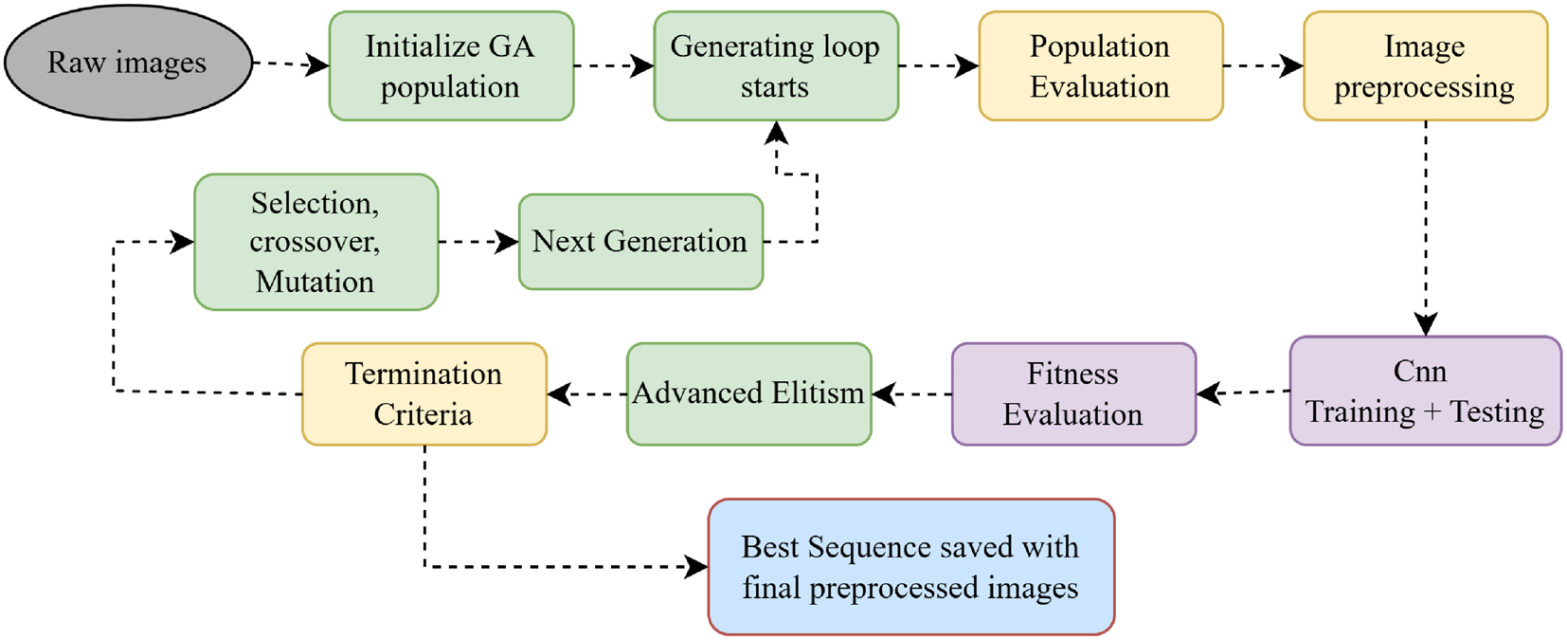
Workflow of genetic algorithm.

The genetic algorithm framework implements advanced evolutionary computation principles for automated pipeline preprocessing optimization, addressing the computational complexity and interdependency challenges inherent in manual optimization approaches. The genetic algorithm incorporates sophisticated mechanisms that include elitism, multi-objective fitness evaluation, and adaptive genetic operators to ensure robust convergence toward optimal preprocessing configurations^37–39^. Table 3 summarizes the complete set of hyperparameters governing the genetic algorithm, including the core evolutionary operators, advanced elitism settings, and sequence structure constraints employed throughout the optimization process (Fig. 8).

**Table 3:** Genetic algorithm hyperparameters.

Genetic algorithm parameters							
Parameter	Population	Generations	$P_c$ (Single-Point)	$P_c$ (Uniform)	$P_m$ (Filter)	$P_m$ (Parameter)	$P_m$ (Length)
Value	20	40	0.8	0.2	0.08	0.20	0.02

Advanced elitism parameters							
Parameter	Hall of fame size		Elite injection		Tournament size		Elite percentage
Value	5		2 per length		3		0.30

Sequence structure							
Parameter	Min sequence length		Max sequence length		Filter pool size		*σ* (Gaussian)
Value	3		6		48		0.1

*Chromosome Encoding and Representation* The genetic algorithm employs variable-length chromosome encoding to represent tunable complexity preprocessing sequences. Each chromosome *C* encodes a preprocessing pipeline consisting of a sequence of filter operations:

1 $$\begin{aligned} C = \left\{ (f_{k_1}, \boldsymbol{\theta }_1), (f_{k_2}, \boldsymbol{\theta }_2), \ldots , (f_{k_L}, \boldsymbol{\theta }_L) \right\} \end{aligned}$$

where:

- $L \in [L_{\min }, L_{\max }]$ denotes the variable sequence length, with $L_{\min } = 3$ and $L_{\max } = 7$ defining the minimum and maximum number of filtering operations
- $f_{k_i}$ represents the *i*-th filter operation, encoded as an integer index $k_i \in \{0, 1, \ldots , 47\}$ corresponding to the comprehensive library of 48 distinct preprocessing operations, including morphological operations, frequency domain filters, edge detection, smoothing, thresholding, and adaptive enhancement techniques
- $\boldsymbol{\theta }_i = [\theta ^{(1)}_i, \theta ^{(2)}_i, \ldots , \theta ^{(m)}_i]^T$ is the parameter vector for filter $f_{k_i}$, where each parameter $\theta ^{(p)}_i$ is a real-valued variable normalized to a specified range $[\theta ^{\min }_i, \theta ^{\max }_i]$ determined by the filter’s operational constraints

This variable-length encoding enables the genetic algorithm to explore preprocessing pipelines of different complexities, allowing evolution to discover optimal trade-offs between feature enhancement, computational cost, and classification robustness across diverse defect types.

***Population Initialization Strategy***

The genetic algorithm initializes the population through random generation, creating 100 individuals where each individual represents a filter sequence. Each sequence is randomly generated by uniformly selecting filter IDs from the 48 available preprocessing filters and randomly assigning parameter values within their valid ranges. The sequence length for each individual is also randomly determined between the minimum (3 filters) and maximum (6 filters) constraints, ensuring diversity in chromosome structure. This random initialization approach ensures comprehensive exploration of the solution space while preventing bias toward any particular filter combination. The population size of 100 individuals provides adequate genetic diversity while maintaining computational feasibility. Using randomness in filter selection, parameter initialization, and sequence length, the algorithm establishes a diverse starting population that enables an effective evolutionary search for optimal preprocessing pipelines in defect detection^40,41^.

***Elitism Mechanism for Solution Preservation***

The genetic algorithm incorporates elitism as a fundamental mechanism to preserve the best-performing preprocessing sequences across generations, preventing the loss of high-quality solutions during the stochastic evolutionary process. Elitism ensures that a predetermined number of elite individuals (typically 5-10% of the population size, corresponding to 5-10 individuals) are directly transferred to the next generation without undergoing crossover or mutation operations.

The implementation of elitism follows established principles in which elite individuals are selected based on their fitness rankings and copied unchanged to the subsequent generation. This strategy serves multiple critical functions:

- Convergence acceleration by maintaining reference points for promising search space regions
- Preservation of solution by preventing the degradation of optimal preprocessing configurations through genetic operations
- Convergence monitoring by tracking the best fitness values across generations to detect optimization plateaus

Elite Selection Strategy: Elite selection employs a fitness-based ranking, where the top performers are identified through a fitness score comparison and are systematically preserved. The elite preservation mechanism selects the best $\varepsilon$ individuals from the current population, where $\varepsilon = 0.05 \times {population}\_size$ (e.g. 5 individuals from 100), ensuring balanced exploitation of high-quality solutions while maintaining population diversity. The elitism strategy addresses the fundamental trade-off between exploitation and exploration by preserving proven solutions while allowing continued search through the remaining population. This approach prevents the common problem of losing optimal solutions due to the stochastic nature of selection, crossover, and mutation operations.

Crossover operations implement specialized techniques for variable-length chromosomes applied only to non-elite individuals: single-point crossover (probability Pc = 0.8) for filter sequence exchange and uniform crossover (Pc = 0.2) for parameter mixing. Elite individuals bypass crossover operations entirely, ensuring that their optimal configurations remain intact across generations.

Mutation Strategies: Mutation strategies incorporate multiple mechanisms applied exclusively to non-elite offspring: filter replacement mutation (probability $P_m = 0.08$) randomly replaces filters with alternatives from the library; parameter mutation ($P_m = 0.2$) applies Gaussian perturbation ($\sigma = 0.1$) to parameter values; and sequence length mutation ($P_m = 0.02$) adds or removes filters from preprocessing sequences. The elitism mechanism protects high-quality solutions from potentially destructive mutations while allowing continued exploration through the remaining population.

Elitist Replacement Strategy: The genetic algorithm implements an evolutionary strategy $(\mu + \lambda )$ where elite individuals from the parent generation compete with newly generated offspring for survival positions. This approach ensures that elite solutions are only replaced if superior alternatives are discovered, maintaining a monotonic improvement in the overall quality of the population.

2 $$\begin{aligned} \textit{fitness}(C) = \alpha \cdot \text {Accuracy}(C) + (1-\alpha ) \cdot \left( 1 - \text {Norm}_{\text {CompCost}}(C)\right) \end{aligned}$$

In Equation (2), $\text {Accuracy}(C)$ denotes CNN classification accuracy on the validation data set obtained from the test model, while $\text {Norm}_{\text {CompCost}}(C)$ represents the normalized computational cost, including preprocessing and inference time, scaled to the range [0,1]. In this article, the weighting parameter $\alpha$ is set to 0.7, balancing the trade-off between accuracy and computational efficiency and effectively prioritizing model accuracy while still accounting for computational cost. This bi-objective formulation ensures that the evolved preprocessing sequences are accurate and computationally practical for industrial deployment, facilitating meaningful comparisons of accuracy and inference time in the experimental results.

The replacement mechanism operates as follows: (1) elite individuals are automatically preserved in the next generation, (2) remaining population slots are filled through competitive selection between non-elite parents and all offspring, and (3) fitness-based ranking determines final population composition while maintaining the predetermined elite count.

Convergence and Termination Criteria: Algorithm termination employs multiple criteria to ensure optimal solution convergence while preventing excessive computational overhead: maximum generation limit (150 generations), fitness improvement threshold (<0.001 over 15 consecutive generations), and maintenance of population diversity through genotypic distance monitoring. The elitism mechanism contributes to the stability of convergence by providing consistent high-quality reference points throughout the evolutionary process.

Convergence analysis incorporates statistical testing for fitness stagnation using the elite population subset, where consistent elite fitness values indicate algorithm convergence toward optimal preprocessing configurations. The elitism mechanism enables reliable convergence detection by maintaining stable performance benchmarks across generations.

This comprehensive integration of elites ensures that the genetic algorithm maintains optimal preprocessing solutions while continuing to explore the search space for potentially superior alternatives, balancing the exploitation and exploration requirements essential for effective evolutionary optimization in image preprocessing applications.

**Algorithm 1:**
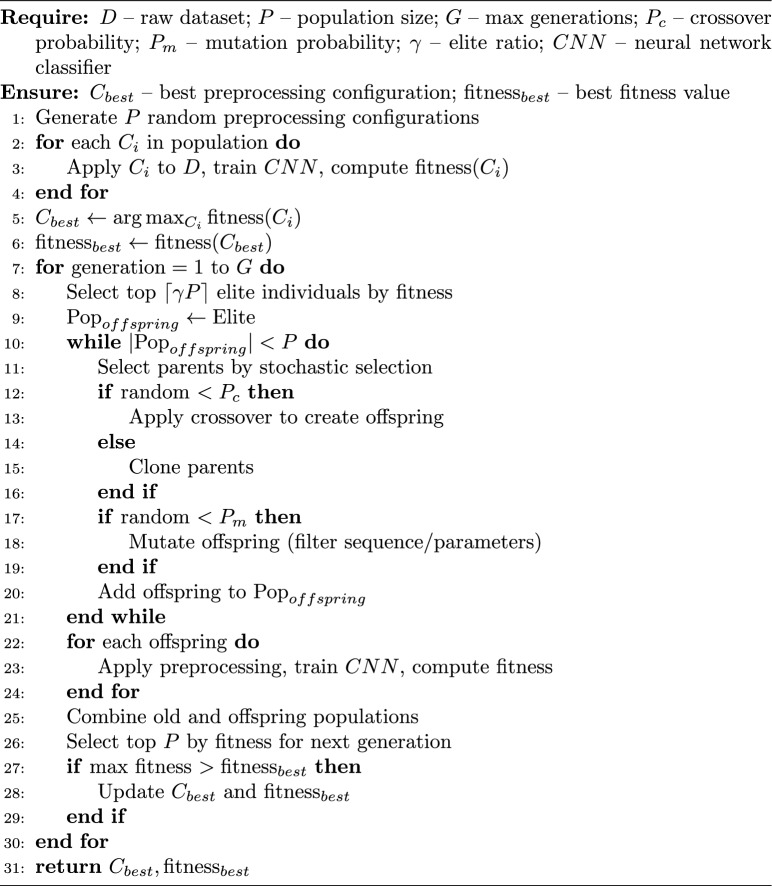
Genetic algorithm for image preprocessing optimization.

**Fig. 9:**
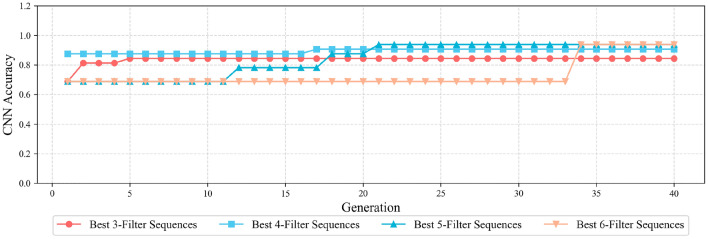
Evolution of CNN accuracy across generations for Product 1.

**Fig. 10:**
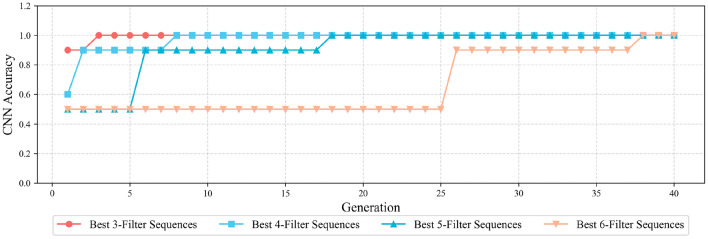
Evolution of CNN accuracy across generations for Product 2.

**Fig. 11:**
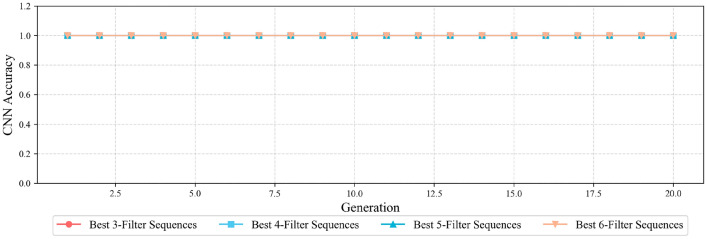
Evolution of CNN accuracy across generations for Product 3.

The graphs provided in Figs. 9, 10, 11 illustrate the evolution of CNN accuracy across generations for genetic algorithm-optimized preprocessing sequences of varying length (3 to 6 filters), evaluated on three different products. For each product, the genetic algorithm rapidly identifies effective filter sequences, with the best-performing configurations achieving significant accuracy improvements within the first 10–15 generations, demonstrating strong convergence behavior. Notably, shorter sequences (3 or 4 filters) often reach optimal or near-optimal accuracy more quickly, whereas longer sequences may require more generations, but sometimes yield incremental gains for more complex datasets.

In Product 1 (Fig. 9) and Product 3 (Fig. 11), several sequence lengths achieve perfect or near-perfect accuracy, while Product 2 (Fig. 10) shows clearer separation between different sequence lengths, highlighting the importance of tuning pipeline complexity to the underlying data. The stability and consistency of the accuracy plateaus across generations reflect the robustness of the genetic algorithm in preserving high-quality solutions through elitism and selection mechanisms. In all cases, the best evolved sequence for each length maintains its performance in later generations, indicating the effectiveness of the algorithm to retain and refine optimal preprocessing strategies over time. These results collectively emphasize the adaptability and efficiency of the genetic algorithm in discovering sequence configurations that maximize neural network performance for diverse industrial image analysis tasks.

***Computational Cost of GA Optimization***

To quantify the computational overhead associated with the evolutionary search process, the total execution time required for the Genetic Algorithm to converge on an optimal preprocessing pipeline was systematically recorded. Across five independent runs, the average computational cost for the GA optimization phase was $391.01 \pm 45.33$ minutes for Product 1, $90.51 \pm 34.13$ minutes for Product 2, and $221.55 \pm 51.38$ minutes for Product 3, where the values are reported as mean ± standard deviation. The variance in total computational time across product categories is primarily attributable to the structural complexity of the defects and the resulting length of the dynamically generated filter sequences required to maximize the fitness function. All GA optimization processes–including population initialization, fitness evaluation, and evolutionary operations–were executed using the hardware and software specifications detailed in Table 4. While the initial offline computational cost of the GA search may be higher or lower than that of manual trial-and-error selection, this represents a one-time, upfront investment. Once the optimal preprocessing sequence is identified, runtime inference during active deployment is highly efficient, as demonstrated by the superior test-phase inference times reported in Section 4.

***Confusion Matrix*** The confusion matrix is a widely used tool in machine learning and statistical analysis to evaluate the performance of classification models^48,49^. Provides a tabular representation of the true labels versus the predicted labels generated by the model. The matrix is particularly helpful for understanding errors made by the classifier and can be used for binary or multiclass classification tasks^50,51^. For a binary classification problem:

- *True Positives (TP):* Correctly predicted positive instances.
- *True Negatives (TN):* Correctly predicted negative instances.
- *False Positives (FP):* Incorrectly predicted positive instances (type I error).
- *False Negatives (FN):* Incorrectly predicted negative instances (type II error)^52^.

**Table 4:** System specifications.

Specification	Detail
Operating system (OS)	Microsoft Windows 11 Pro
Processor	13th Gen Intel(R) Core(TM) i7-1360P @ 2.20 GHz
RAM	16.0 GB
Storage	1 TB SSD
System architecture	64-bit operating system, x64-based processor
Motherboard manufacturer	ASUSTeK COMPUTER INC.

### Advantages of genetic algorithm optimization

- Global Search Capability: Explores multiple solution regions simultaneously, avoiding local optima through population diversity and stochastic operators.
- Automated Parameter Selection: Eliminates manual preprocessing parameter tuning, reducing expert dependency, and enabling automatic filter sequence optimization.
- Multi-Objective Optimization: Simultaneously optimizes detection accuracy, computational efficiency, and robustness through weighted multi-objective fitness functions.

### Limitations of genetic algorithm optimization

- High Computational Cost: Requires extensive resources for fitness evaluations across populations, significantly increasing processing time and hardware requirements.
- Premature Convergence Risk: May converge to suboptimal solutions when genetic diversity is lost, providing no theoretical optimality guaranties.
- Parameter Sensitivity Issues: Performance is highly dependent on control parameter settings, requiring extensive experimentation and domain expertise for optimization.

#### Sequences generated by genetic algorithm

In the process of optimizing the preprocessing pipelines with a genetic algorithm, a diverse set of candidate sequences was automatically generated, ranging from three-filter to six-filter configurations. The filters available for selection are listed in Table 2, while their corresponding parameter bounds (lower and upper limits) are defined in the YAML-style configuration provided in the Appendix. This approach allowed the GA to rigorously explore a wide range of filter combinations and parameter settings, maximizing the search for sequences that best fit the neural network in defect detection. Each sequence was evaluated using a robust fitness function that emphasizes classification performance and operational efficiency, ensuring that both precision and practical applicability were considered during selection.

From the collection of candidate sequences, the final sequence chosen for detailed comparison is the one that demonstrated the highest validation accuracy during the evolutionary optimization process. This selection strategy ensures that the reported results represent the optimal achievable benefit of automated pipeline optimization for the respective data set and model architecture. By presenting the highest-performing sequence, the analysis highlights the effectiveness of the genetic algorithm in adaptively identifying preprocessing strategies that outperform standard or manually tuned pipelines, providing a meaningful benchmark for industrial machine vision applications.

***Product 1***

*Sequence: (37, 42, 33, 0, 3, 40)*

The Earliest best preprocessing sequence for Product 1 was found in generation 34, 6-filter sequence**(37, 42, 33, 0, 3, 40)**, corresponding to the following filters and parameters:

- **histogram qualification** (37)
- **gaussian filter**(42) with a sigma value of **3.931298600338181**
- **closing operation** (33) with a Kernel size of **3**
- **Binarization** (0) with a threshold value of **165**
- **Enchanced image** (3)
- **median filter** (40) with a Kernel size of **9**

***Product 2***

*Sequence: (16, 0, 6)*

The Earliest best preprocessing sequence for Product 1 was found in generation 3, 3-filter sequence**(16, 0, 6)**, corresponding to the following filters and parameters:

- **Average filter** (16)
- **Binarization** (0) with a threshold value of **47**
- **smoothing noise** (6) with a Kernel size of **3**

***Product 3***

*Sequence: (0, 6, 42)*

The Earliest best preprocessing sequence for Product 1 was found in generation 1, 3-filter sequence**(0, 6, 42)**, corresponding to the following filters and parameters:

- **Binarization ** (0) with a threshold value of **140**
- **smoothing noise** (6)
- **gaussian filter**(42) with a threshold value of **76**

### Neural network modelling

**Fig. 12:**
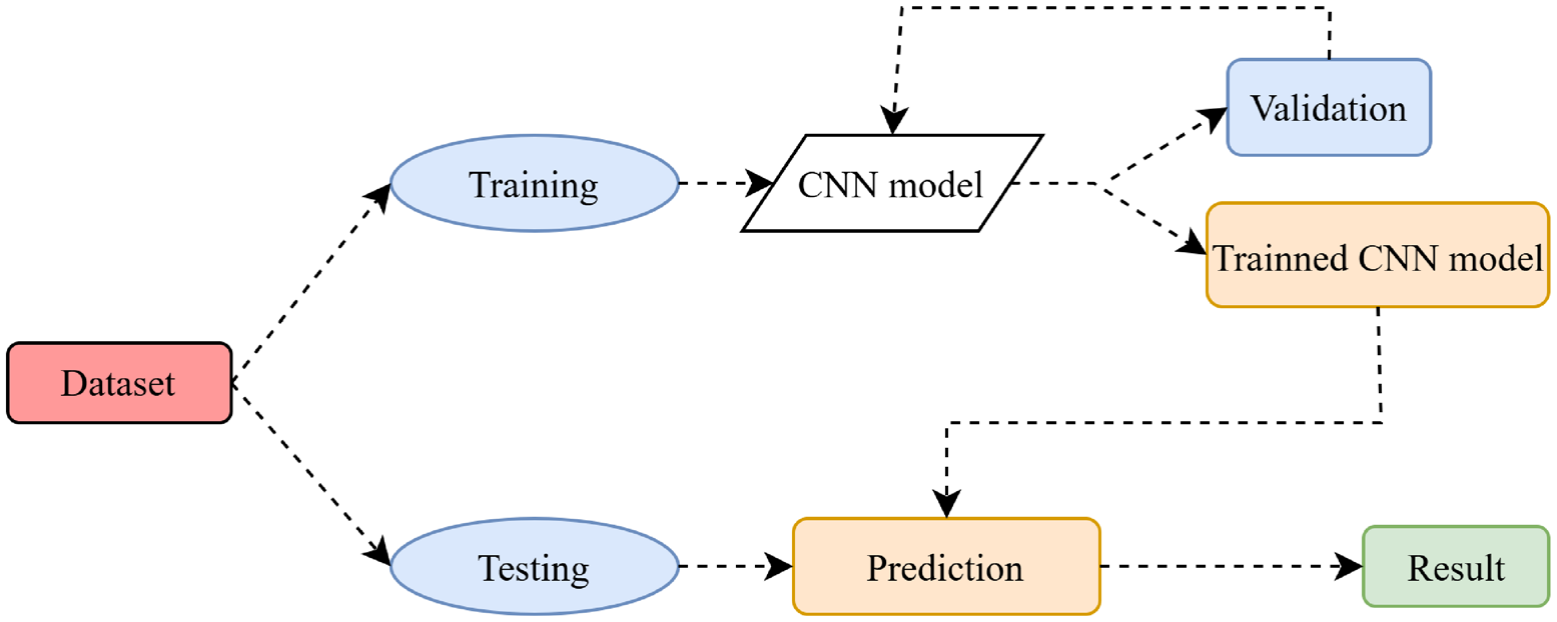
The flowchart to implementation of the CNN model.

A neural network is a computational system inspired by the structure and function of the human brain. It consists of layers of interconnected nodes (neurons) that process data through weighted connections. Neural networks are particularly effective in tasks that require pattern recognition, such as image classification, object detection, and speech recognition. In this research, a convolutional neural network (CNN) was used due to its suitability for image-related tasks. CNNs are specifically designed to process and analyze visual data by leveraging spatial hierarchies in images^42,43^. This study used two sets of images: the original images and the preprocessed images. The preprocessed images were generated using three distinct preprocessing methodologies (Fig. 12).

For each configuration, the individually trained models were evaluated to determine the accuracy achieved and the minimum number of images required to achieve satisfactory training for both the original and preprocessed images. Furthermore, the results were analyzed to compare the performance of models trained with different preprocessing sequences with those trained on the original images.

### Convolutional neural network (CNN)

Convolutional Neural Networks (CNNs) are widely used for image analysis and defect detection due to their ability to automatically extract hierarchical features from images^44,45^. The complete architecture and training configuration of the model are summarized in Table 5. Applying convolutional layers, CNNs detect edges, textures, and patterns, making them highly effective for classification and anomaly detection tasks. The network is trained on labeled datasets, allowing it to learn optimal feature representations and improve accuracy over time. When integrated with optimized preprocessing techniques, CNNs improve defect recognition by focusing on the most relevant image features while minimizing noise and artifacts.

**Table 5:** CNN architecture and training configuration.

CNN architecture	Training configuration
Input: $256 \times 256 \times 1$ (grayscale images)	Optimizer: Adamax
Conv2D (16 filters, $4 \times 4$, same padding)	Learning rate: 0.0001
MaxPooling ($4 \times 4$)	Loss: Sparse Categorical Crossentropy
Conv2D (32 filters, $4 \times 4$, same padding)	Batch size: 4
MaxPooling ($4 \times 4$)	Epochs: 40
Conv2D (64 filters, $2 \times 2$, same padding)	Train/Test split: 80% / 20%
MaxPooling ($2 \times 2$)	Metric: Accuracy
Conv2D (128 filters, $2 \times 2$, same padding)	Learning rate scheduler: ReduceLROnPlateau
Conv2D (128 filters, $2 \times 2$)	Patience: 100 epochs
MaxPooling ($2 \times 2$)	Model checkpoint: every 50 batches
Conv2D (256 filters, $2 \times 2$)	Data normalization: Pixel scaling to [0,1]
Conv2D (256 filters, $2 \times 2$)	Data augmentation: None
MaxPooling ($2 \times 2$)	Early stopping: Not applied
Flatten	Dropout: 0.3
Dense (128, ReLU)	
Dense (64, ReLU)	
Dense (32, ReLU)	
Dense (16, ReLU)	
Output Dense (Softmax, C classes)	

### Evaluation metrics

The evaluation of the neural network model is performed using a set of key parameters that provide information on the performance and efficiency of the model. These parameters play a crucial role in deciding the best order of the preprocessing steps and determining the number of images needed to ensure successful model training. Metrics such as accuracy, sensitivity, specificity, precision, execution time, memory usage, and the confusion matrix are systematically analyzed to assess the model’s ability to achieve reliable and efficient results^46,47^. By carefully examining these metrics, it becomes possible to identify the most suitable preprocessing pipeline and the ideal data set size for training, ensuring that the model achieves optimal performance while maintaining computational efficiency. This structured evaluation process is critical for selecting the best configuration for specific applications and use cases.

## Results

This section evaluates the performance of the proposed convolutional neural network (CNN) across three distinct product categories. The evaluation systematically compares the raw-image baseline, manually engineered preprocessing pipelines, and the proposed Genetic Algorithm (GA) optimization framework. Performance is assessed using classification metrics (accuracy, precision, sensitivity, and specificity) and computational efficiency (inference and preprocessing times). The objective is to demonstrate the impact of automated preprocessing optimization on both classification reliability and operational speed.

### Comparative performance analysis

***Product 1***

**Fig. 13:**
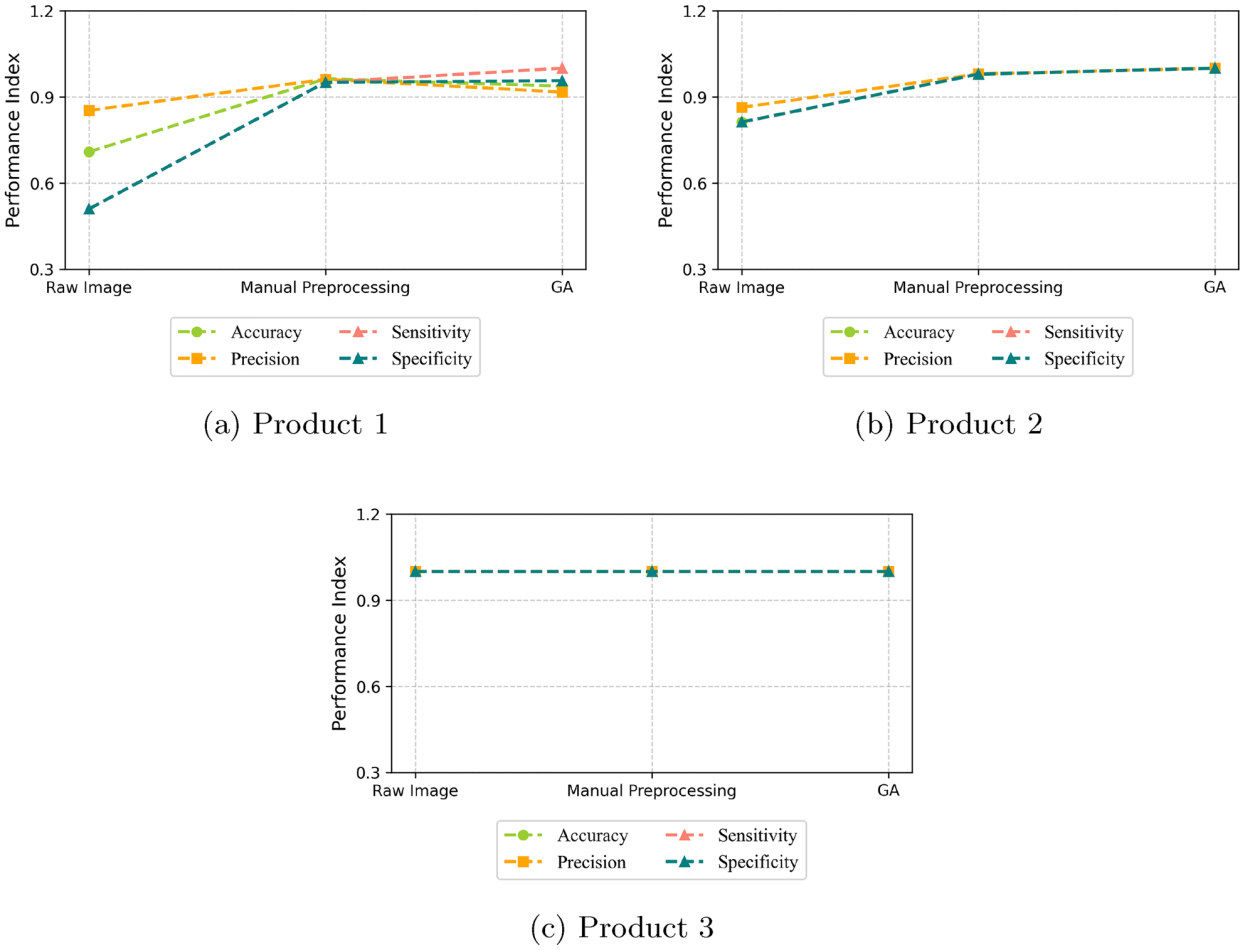
Performance metrics for three product types: (**a**) Product 1, (**b**) Product 2, and (**c**) Product 3.

As detailed in Table 6, the raw-image baseline for Product 1 struggled with feature extraction, resulting in poor classification sensitivity. Both preprocessing interventions substantially improved overall performance. Although the manual preprocessing pipeline achieved a marginally higher peak accuracy, the GA-optimized sequence delivered highly comparable classification results (Fig. 13a) while maximizing computational efficiency. Specifically, the GA framework recorded the fastest inference time, successfully balancing high accuracy with reduced latency, proving the viability of automated sequence generation over manual tuning. The performance metrics of the baseline and manual preprocessing methods for Product 1 are presented in Tables 7 and 8, respectively.

**Table 6:** Comparative performance analysis - fixed 80/20 split for Product 1.

Parameter	Baseline (raw images)	Manual preprocessing	Genetic algorithm
Inference time (s)	2.296	1.9375	1.886
PR time (s)	0	58.22	0
Total time (s)	2.296	60.16	1.886
Accuracy	0.7088	0.963	0.9375
Precision	0.853	0.9614	0.9167
Sensitivity	0.510	0.9509	1
Specificity	0.510	0.9509	0.9565

PR time indicates image Preprocessing Techniques time, for Baseline performance it is 0 Because the model trained and tested on raw images

**Table 7:** Baseline performance metrics - raw image processing (Product 1).

Metric	8	16	24	32	40	48	52
inference time (s)	1.57	1.88	1.95	2.10	2.19	2.27	2.39
PR time (s)	0	0	0	0	0	0	0
Total (s)	1.57	1.88	1.95	2.10	2.19	2.27	2.39
Accuracy	0.600	0.685	0.564	0.585	0.607	0.780	0.875
Precision	0.845	0.782	0.780	0.440	0.480	0.780	0.780
Sensitivity	0.583	0.810	0.638	0.611	0.627	0.645	0.805
Specificity	0.583	0.810	0.638	0.611	0.627	0.645	0.805
Memory (MB)	309.5	318.6	291.5	335.2	340.4	341.0	393.3

**Table 8:** Manual preprocessing sequence (Product 1).

Metric	8	16	24	32	40	48	52
Inf. Time (s)	0.977	1.359	1.437	1.813	1.719	1.562	1.609
PR Time (s)	42.53	76.41	126.1	161.9	199.7	237.0	240.6
Total (s)	43.51	77.77	127.5	163.7	201.4	238.6	242.2
Accuracy	0.687	0.879	0.919	0.929	0.919	0.949	1.000
Precision	0.801	0.878	0.923	0.930	0.923	0.949	1.000
Sensitivity	0.702	0.878	0.917	0.931	0.922	0.950	1.000
Specificity	0.702	0.878	0.917	0.931	0.922	0.950	1.000
Memory (MB)	139.5	188.9	188.9	231.3	231.6	242.3	259.9

PR Time refers to preprocessing time, which is the duration required to apply the pipeline of methods to the datasets. Inf. Time = Inference Time

***Product 2***

The evaluation of Product 2, summarized in Table 9, highlighted the robust features extraction capabilities of evolutionary optimization. The GA-optimized pipeline outperformed both manually selected filters and baseline in primary classification metrics. As visualized in the confusion matrices (Fig. 13b), the manual approach, while an improvement over the baseline, exhibited inconsistencies in precision. In contrast, the GA method effectively stabilized classification performance while simultaneously reducing inference time, demonstrating its superiority in handling this specific product’s defect morphologies.The performance metrics of the baseline and manual preprocessing methods for Product 2 are presented in Tables 10 and 11, respectively.

**Table 9:** Comparative performance analysis - Fixed 80/20 Split for Product 2.

Parameter	Baseline (raw images)	Manual preprocessing	Genetic algorithm
Inf. time (s)	1.2187	1.02	1.299
PR time (s)	0	66.5	0
Total time (s)	1.2187	67.52	1.299
Accuracy	0.8125	0.9791	1
Precision	0.8636	0.98	1
Sensitivity	0.8125	0.9791	1
Specificity	0.8125	0.9791	1

**Table 10:** Baseline performance metrics - raw image processing (Product 2).

Metric	8	12	16	20	24
Inf. time (s)	0.828	0.875	0.890	0.921	1.297
PR time (s)	0	0	0	0	0
Total (s)	0.828	0.875	0.890	0.921	1.297
Accuracy	0.854	0.500	0.875	0.958	0.917
Precision	0.887	0.750	0.900	0.961	0.929
Sensitivity	0.854	0.500	0.875	0.958	0.917
Specificity	0.854	0.500	0.875	0.958	0.917
Memory (MB)	215.8	216.5	224.9	230.1	244.8

**Table 11:** Manual preprocessing sequence (Product 2).

Metric	8	12	16	20	24
Inf. Ttme (s)	1.093	1.125	1.140	1.156	1.140
PR time (s)	10.8	16.62	21.9	33.03	35.49
Total (s)	11.893	17.745	23.04	34.186	36.63
Accuracy	0.854	0.854	0.938	0.938	1.000
Precision	0.855	0.855	0.944	0.944	1.000
Sensitivity	0.854	0.854	0.938	0.938	1.000
Specificity	0.854	0.854	0.938	0.938	1.000
Memory (MB)	139.5	188.9	188.9	231.3	231.6

PR Time refers to preprocessing time, which is the duration required to apply the pipeline of methods to the datasets. Inf. Time = Inference Time

***product 3***

Unlike the previous categories, Product 3 features highly distinguishable defect characteristics. As shown in Table 12, this allowed all three methodologies–including the baseline–to achieve optimal or near-optimal classification accuracy (approaching 100%). Because accuracy was saturated (Fig. 13c), the primary comparative metric for this product was computational efficiency. The GA-optimized pipeline proved highly advantageous in this regard, delivering the shortest inference times and entirely eliminating the extensive manual preprocessing overhead required by the expert-designed pipeline.

**Table 12:** Comparative performance analysis for Product 3.

Parameter	Baseline (raw images)	Manual preprocessing	Genetic algorithm
Inf time (s)	2.25	2.07	1.988
PR time (s)	0	39.88	0
Total time (s)	2.25	41.95	1.988
Accuracy	1	1	1
Precision	1	1	1
Sensitivity	1	1	1
Specificity	1	1	1

**Table 13:** Baseline performance metrics - raw image processing (Product 3).

Metric	8	16	24	32	40	48	60
Inf. time (s)	1.87	2.07	2.156	2.171	2.218	2.25	2.25
PR time (s)	0	0	0	0	0	0	0
Total (s)	1.87	2.07	2.156	2.171	2.218	2.25	2.25
Accuracy	0.989	0.978	1.000	0.998	1.000	1.000	0.993
Precision	0.989	0.956	1.000	0.994	1.000	1.000	0.995
Sensitivity	0.967	0.983	1.000	0.983	1.000	1.000	0.983
Specificity	0.967	0.983	1.000	0.983	1.000	1.000	0.983
Memory (MB)	312.1	335.0	343.4	350.6	354.0	366.0	377.3

**Table 14:** Manual preprocessing sequence (Product 3).

Metric	8	16	24	32	40	48	60
Inf. time (s)	1.781	1.468	1.656	1.578	1.703	1.625	1.718
PR time (s)	10.99	21.6	35.44	45.99	55.69	67.71	82.71
Total (s)	12.771	23.068	37.096	47.568	57.393	69.335	84.428
Accuracy	1.000	1.000	0.983	0.983	1.000	1.000	1.000
Precision	1.000	1.000	0.984	0.984	1.000	1.000	1.000
Sensitivity	1.000	1.000	0.982	0.982	1.000	1.000	1.000
Specificity	1.000	1.000	0.982	0.982	1.000	1.000	1.000
Memory (MB)	224.3	228.7	229.1	232.4	234.2	239.6	252.9

PR Time refers to preprocessing time, which is the duration required to apply the pipeline of methods to the datasets. Inf. Time = Inference Time

### Impact of training dataset size and classification balance

To evaluate the data efficiency of the respective preprocessing methodologies, CNN models were trained across systematically varied dataset sizes, ranging from 8 to 60 images per class. The incremental performance metrics are documented in Tables 7 and 8 (Product 1), Tables 10 and 11 (Product 2), and Tables 13 and 14 (Product 3). Across all three product categories, the baseline models required significantly larger training sets to reach acceptable performance thresholds. In contrast, the application of GA-optimized preprocessing accelerated model convergence, requiring 30% to 60% fewer training images to achieve target accuracies. Although manual preprocessing also improved data efficiency relative to baseline, it exhibited greater performance variability at lower sample sizes.

**Fig. 14:**
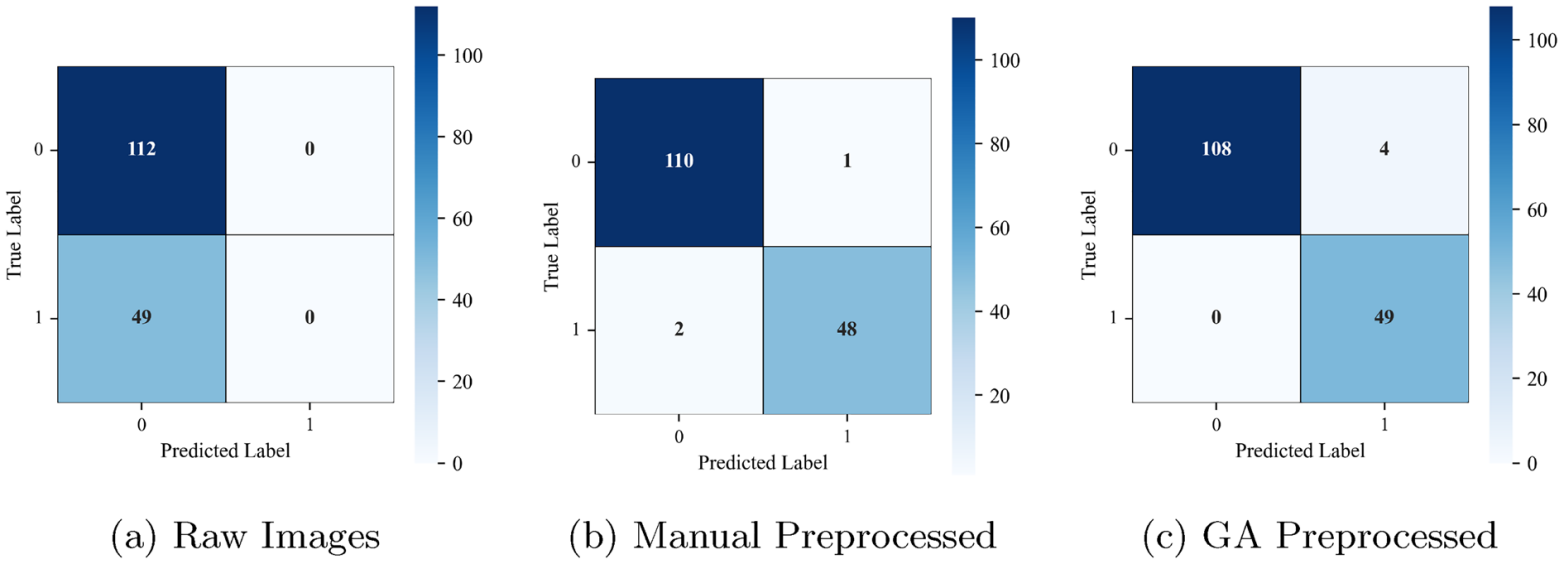
Confusion matrices for Product 1: (**a**) Raw images baseline; (**b**) Manual preprocessing method; (**c**) Genetic Algorithm preprocessing method.

**Fig. 15:**
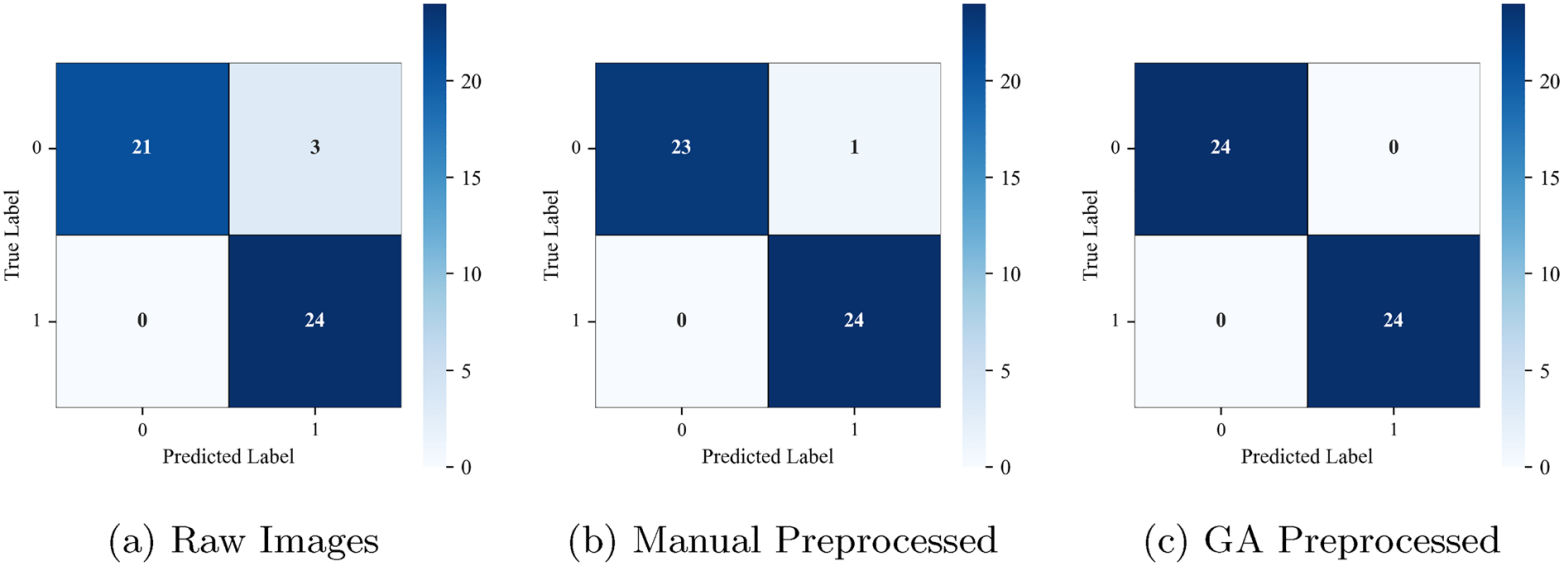
Confusion matrices for Product 2: (**a**) Raw images baseline; (**b**) Manual preprocessing method; (**c**) Genetic Algorithm preprocessing method.

**Fig. 16:**
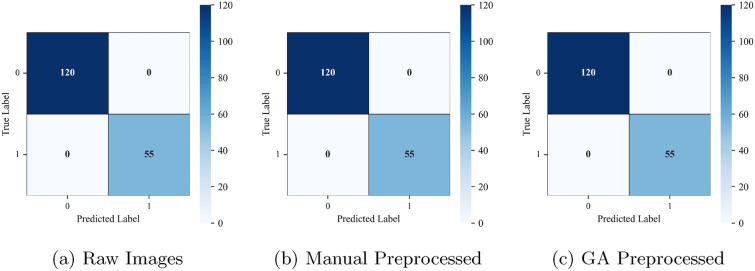
Confusion matrices for Product 3: (**a**) Raw images baseline; (**b**) Manual preprocessing method; (**c**) Genetic Algorithm preprocessing method.

Furthermore, the stability of this classification performance is visually corroborated by the confusion matrices for Product 1 (Fig. 14), Product 2 (Fig.15) and Product 3 (Fig. 16). Across all three products, the baseline raw images frequently struggled with false positives and false negatives, particularly in lower-data scenarios where noise easily confused the network. Although manual preprocessing pipelines reduced these errors, GA-optimized sequences consistently yielded the most balanced true positive and true negative rates. By successfully enhancing the most discriminative image features and suppressing irrelevant noise, the evolutionary optimization framework minimizes class confusion, thereby reducing the neural network’s dependency on large, manually annotated datasets.

*Analysis of product-specific performance and preprocessing strategies* The experimental results demonstrate that the optimal preprocessing strategy varies significantly depending on the inherent visual characteristics, the acquisition environment, and the morphological diversity of the defects. A key factor driving the performance divergence in this study is the consistency of the image data. The dataset for Product 3 was captured directly within an active industrial manufacturing environment with highly controlled lighting. Furthermore, the types of defects in Product 3 are highly consistent and repetitive. Because the visual variance is minimal and heavy feature extraction was not required, the GA adapted by optimizing for efficiency. It successfully converged on shorter, computationally lightweight filter sequences that drastically reduced the number of training images required to reach peak performance, while also maintaining lower inference times compared to the manual approach.

In contrast, the datasets for Products 1 and 2 were acquired in a laboratory setup where environmental variables were less strictly controlled, introducing surface noise. For Product 1, this challenge is compounded by high intra-class variance; the dataset contains many completely different types of defects, but with fewer representative images for each specific type. Manually engineering a single preprocessing pipeline that successfully generalizes across all these diverse defect morphologies is highly complex. The GA successfully addressed this by evaluating the dataset holistically and identifying a universally effective sequence–prioritizing noise smoothing and low-pass filtering. This global strategy effectively suppressed variable background noise while preserving the heterogeneous defect boundaries, allowing the CNN to maintain high accuracy despite the lack of defect uniformity.

Based on the evolutionary patterns observed across these datasets, we can provide specific guidance for deploying machine vision systems in environments with noisy images or highly variable defect types. When a single preprocessing methodology must be applied to a product with multiple distinct defect morphologies and substantial noise, optimization frameworks should prioritize spatial filtering (e.g., Gaussian or median filters) early in the pipeline. This prevents the amplification of artifacts that might mimic distinct defects. Furthermore, the GA should be permitted to explore longer sequence lengths, as a multi-step approach of smoothing followed by targeted, generalized enhancement is required to isolate true regions of interest across diverse defect patterns without introducing false positives.

### Performance variability and statistical significance

Table 15 reports the descriptive statistics for all three preprocessing methodologies under the fixed 80/20 stratified train–test split, averaged over five independent runs. For each product and method, we present the mean and standard deviation of accuracy, precision, sensitivity and specificity. These results summarize the central tendency and variability of model performance across repeated experiments, and provide a stable estimate of how baseline, manual preprocessing, and genetic algorithm–based preprocessing behave under identical data conditions.

**Table 15:** Mean (±SD) performance over 5 runs for each product and preprocessing method under the fixed 80/20 split.

Product	Method	Accuracy	Precision	Specificity	Sensitivity
P1	Baseline	0.794 (±0.0167)	0.724 (±0.0236)	0.700 (±0.186)	0.492 (±0.0396)
	Manual	0.976 (±0.017)	0.976 (±0.017)	0.97 (±0.02)	0.97 (±0.02)
	GA	0.8937 (±0.047)	0.88 (±0.065)	0.893 (±0.044)	0.0893 (±0.052)
P2	Baseline	0.654 (±0.217)	0.814 (±0.094)	0.654 (±0.217)	0.5527 (±0.305)
	Manual	0.876 (±0.0681)	0.88 (±0.0658)	0.879 (±0.0681)	0.878 (±0.0686)
	GA	1.00 (±0.0)	1.00 (±0.00)	1.00 (±0.00)	1.00 (±0.0)
P3	Baseline	1.00 (±0.00)	1.00 (±0.00)	1.00 (±0.00)	2.25 (±0.03)
	Manual	1.00 (0.00)	1.00 (0.00)	1.00 (0.00)	1.00 (0.00)
	GA	1.00 (0.00)	1.00 (0.00)	1.00 (0.00)	1.00 (0.00)

Values are mean (standard deviation) over five independent runs with identical stratified 80/20 train–test partitions across methods

**Table 16:** One-sided Wilcoxon signed-rank test *p*-values comparing accuracy across 5 independent runs.

Product	GA > baseline	GA > manual	Manual > baseline
P1	0.156	1.000	0.062
P2	0.031*	0.031*	0.062
P3	1.000	0.250	1.000

A *p*-value $< 0.05$ indicates a statistically significant improvement (marked with *). The minimum mathematically possible *p*-value for a one-sided Wilcoxon test with $n=5$ is 0.031, which is achieved when a method consistently outperforms the other across all trials

Table 16 presents the statistical significance of accuracy improvements between the preprocessing methodologies, evaluated using a one-sided Wilcoxon signed-rank test ($\alpha = 0.05$) across the five independent runs. For Product 2, the genetic algorithm (GA) achieved a statistically significant improvement in classification accuracy over both the raw-image baseline ($p = 0.031$) and the manually configured pipeline ($p = 0.031$). For Product 1, while GA and manual preprocessing performed similarly, manual preprocessing showed a strong trend toward outperforming the baseline ($p = 0.062$), constrained only by the mathematical bounds of the small sample size ($n=5$). For Product 3, all methods achieved optimal or near-optimal accuracy, rendering differences statistically non-significant ($p \ge 0.250$); thus, the primary advantage of the GA approach for this product lies in its computational efficiency rather than accuracy gains.

### Limitation of the study

While the proposed genetic algorithm-based preprocessing framework demonstrates significant improvements over baseline and manual methods, several contextual limitations must be explicitly acknowledged. First, the experimental validation is confined to a proprietary dataset comprising three specific product categories. Although these categories exhibit distinct defect characteristics, they may not fully represent the extreme variability found across all industrial manufacturing environments (e.g., severe lighting fluctuations, overlapping multi-defect instances, or highly reflective surfaces). Consequently, claims regarding the broad scalability and universal applicability of this method should be interpreted within the parameters of the evaluated image types. Second, as discussed in Section 3.2, the computational cost of the GA optimization phase is non-trivial (ranging from 90 to 391 minutes per product). While this is a one-time offline cost, it may pose challenges in environments requiring continuous, real-time adaptation to rapidly changing product lines without access to high-performance computing infrastructure. Finally, the framework relies on a predefined library of 48 traditional preprocessing filters; the performance upper bound is therefore inherently constrained by the capabilities of these constituent filters. Future studies expanding the validation to larger, public industrial benchmark datasets (e.g., MVTec AD) and incorporating deep-learning-based preprocessing modules will be necessary to further establish the method’s generalizability.

## Conclusion

This study successfully developed and evaluated image preprocessing methodologies tailored for machine vision neural network applications. These methods support key functionalities such as defective (D) and non-defective (ND) quality detection as well as fault localization.

The research focused on analyzing various preprocessing techniques, assessing the impact of different parameters on machine vision performance, and interpreting their influence. Using Genetic Algorithm optimization, several preprocessing pipelines were proposed. These pipelines were implemented, tested, and compared with original (non pre-processed) images through a systematic evaluation of performance metrics.

**Fig. 17:**
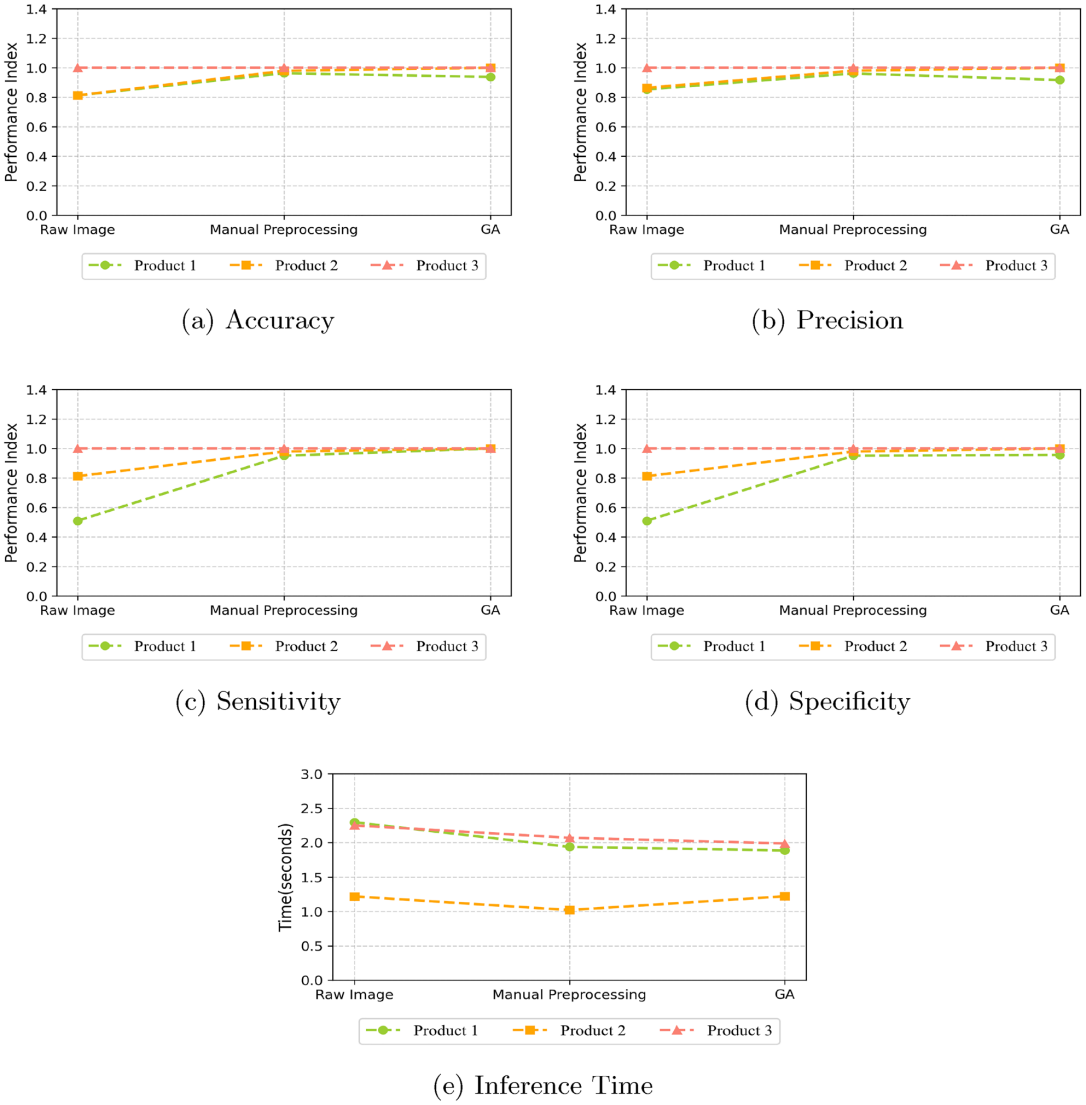
Performance metrics comparison across all products and preprocessing methods: (**a**) Accuracy; (**b**) Precision; (**c**) Sensitivity; (**d**) Specificity; (**e**) Inference Time.

Figure 17a–d, illustrate a comparative overview of performance in all three product categories using the fixed data split. The results reveal that genetic algorithm–based preprocessing consistently outperformed both baseline and trial-and-error preprocessing methods in all key performance indicators, including precision, precision, sensitivity and specificity. Although the trial-and-error approach achieved results closely aligned with the genetic algorithm, it still showed slight variations under certain conditions, reaffirming the adaptive advantage provided by evolutionary optimization strategies in parameter selection and filter sequencing.

Regarding computational efficiency, the genetic algorithm demonstrated a significantly shorter inference time in most test cases, emphasizing its ability to achieve high classification accuracy without compromising processing speed. In some instances, its execution time was close to that of manually optimized sequences while maintaining superior predictive performance. It is important to note that the execution times reported in the comparative Fig. 17e represent only the inference time required for model prediction once the optimal preprocessing sequence has been identified. The complete optimization process involves substantially different time investments for each methodology: manual trial-and-error preprocessing requires extensive iterative experimentation ranging from approximately 10 minutes to several days depending on the complexity of parameter search and the number of filter combinations tested, whereas genetic algorithm optimization time is determined by the selected hyperparameters (population size and generation limit), typically completing within a few hours while systematically exploring the entire search space.

This distinction highlights a critical advantage of the genetic algorithm approach: while both methods ultimately achieve comparable inference performance, the automated evolutionary optimization process provides systematic, reproducible, and comprehensive search space exploration without requiring human expertise or subjective decision-making during the optimization phase. The ability of the genetic algorithm to parallelize fitness evaluations and maintain consistent optimization protocols across different product categories further enhances its practical applicability for industrial deployment, where rapid adaptation to new types of defects and product variations is essential. In general, these results confirm that the evolutionary optimization process led by the genetic algorithm provides a more balanced and scalable solution, ensuring both precision and efficiency in defect detection in various industrial scenarios while significantly reducing the human effort and time required for preprocessing pipeline design.

While the GA-based framework demonstrated significant advantages over manual preprocessing, an important limitation of the current study is the lack of empirical benchmarking against other advanced optimization paradigms. Future work will address this gap by conducting comprehensive comparisons between the proposed GA method and alternative meta-heuristic approaches, including discrete variants of Particle Swarm Optimization (PSO) and advanced Bayesian Optimization strategies adapted for mixed-variable search spaces. Moreover, exploring the integration of our framework with emerging computer vision AutoML pipelines could offer a promising direction for generalized automated preprocessing-pipeline generation across a broader range of industrial settings.

## Data Availability

*Publicly available repository*: The datasets used in this study to support the findings of **Product 1** and **Product 3** are publicly accessible at: https://github.com/challasaiprakash/Image_preprocessing_data/blob/main/raw_data.zip. The parameter configurations, including the upper and lower bounds employed in the Genetic Algorithm optimization, are available at: https://github.com/challasaiprakash/Image_preprocessing_data/blob/main/Parameters_and_bonds.json. *Restricted access*: Data for **Product** **2** are not publicly available due to commercial confidentiality and contractual obligations with the data provider. These data were used under license for the current study, but may be available upon reasonable request, subject to approval from the data provider.

## References

[1] Chan, S. et al. Feature optimization-guided high-precision and real-time metal surface defect detection network. *Sci. Rep.* **14**(1), 31941. 10.1038/s41598-024-83430-3 (2024). 39738521 10.1038/s41598-024-83430-3 PMC11685404

[2] Hou, M. et al. CNN-based defect detection in manufacturing. *Adv. Control Appl.* **6**(4), e196. 10.1002/adc2.196 (2024).

[3] Liu, B. et al. Industrial printing image defect detection using multi-edge feature fusion algorithm. *Complexity* **2021**(1), 2036466. 10.1155/2021/2036466 (2021).

[4] Tsai, D. M. & Rivera Molina, D. E. Morphology-based defect detection in machined surfaces with circular tool-mark patterns. *Measurement* **134**, 209–217. 10.1016/j.measurement.2018.10.079 (2019).

[5] Hussain, W. et al. Ensemble genetic and CNN model-based image classification by enhancing hyperparameter tuning. *Sci. Rep.* **15**(1), 1003. 10.1038/s41598-024-76178-3 (2025). 39762285 10.1038/s41598-024-76178-3 PMC11704345

[6] Khanam, R. et al. A comprehensive review of convolutional neural networks for defect detection in industrial applications. *IEEE Access* **12**, 94250–94295. 10.1109/ACCESS.2024.3425166 (2024).

[7] Sun, Y. et al. Automatically designing CNN architectures using the genetic algorithm for image classification. *IEEE Trans. Cybern.* **50**(9), 3840–3854. 10.1109/TCYB.2020.2983860 (2020). 32324588 10.1109/TCYB.2020.2983860

[8] Taha, Z. Y., Abdullah, A. A. & Rashid, T. A. Optimizing feature selection with genetic algorithms: A review of methods and applications. *Knowl. Inf. Syst.*10.1007/s10115-025-02515-1 (2025).

[9] Zafar, A. et al. An optimization approach for convolutional neural network using non-dominated sorted genetic algorithm-II. *Comput. Mater. Continua* **74**(3), 5641–5661. 10.32604/cmc.2023.033733 (2023).

[10] Hami, M. & JameBozorg, M. Assessing The Impact of CNN Auto Encoder-Based Image Denoising on Image Classification Tasks. arXiv:2404.10664 [cs] (2024).

[11] Nguyen, H., Nguyen, T. A. & Toan, N. D. Optimizing feature extraction and fusion for high-resolution defect detection in solar cells. *Intell. Syst. Appl.* **24**, 200443. 10.1016/j.iswa.2024.200443 (2024).

[12] Rodrigues, L. F., Naldi, M. C. & Mari, J. F. Comparing convolutional neural networks and preprocessing techniques for HEp-2 cell classification in immunofluorescence images. *Comput. Biol. Med.* **116**, 103542. 10.1016/j.compbiomed.2019.103542 (2020). 31790962 10.1016/j.compbiomed.2019.103542

[13] Sioma, A. Assessment of wood surface defects based on 3D image analysis. *Wood Res.* **60** (2015).

[14] Hao, Z. et al. Surface defect segmentation algorithm of steel plate based on geometric median filter pruning. *Front. Bioeng. Biotechnol.* **10**. 10.3389/fbioe.2022.945248 (2022). 10.3389/fbioe.2022.945248 PMC9283705 35845429

[15] Guan, S. et al. Adaptive median filter salt and pepper noise suppression approach for common path coherent dispersion spectrometer. *Sci. Rep.* **14**, 17445. 10.1038/s41598-024-66649-y (2024). 39075128 10.1038/s41598-024-66649-y PMC11286951

[16] Gong, Y. et al. A transfer learning object detection model for defects detection in X-ray images of spacecraft composite structures. *Compos. Struct.* **284**, 115136. 10.1016/j.compstruct.2021.115136 (2022).

[17] Wang, X. & Yu, X. Understanding the effect of transfer learning on the automatic welding defect detection. *NDT & E Int.* **134**, 102784. 10.1016/j.ndteint.2022.102784 (2023).

[18] Lin, H. D., Wu, H. L. & Lin, C. H. A deep transfer learning-based visual inspection system for assembly defects in similar types of manual tool products. *Sensors* **25**(6), 1645. 10.3390/s25061645 (2025). 40292690 10.3390/s25061645 PMC11945499

[19] Qureshi, F., Sharma, S. & Nazir, R. Metaheuristics developing intelligent solutions for complex optimization problems. In *Metaheuristic Algorithms and Optimizing Neural Networks for Biomedical Image Processing* 37–66. 10.4018/979-8-3373-0523-3.ch002. https://www.igi-global.com/chapter/metaheuristics-developing-intelligent-solutions-for-complex-optimization-problems/www.igi-global.com/chapter/metaheuristics-developing-intelligent-solutions-for-complex-optimization-problems/387488 (IGI Global Scientific Publishing, 2026).

[20] Wang, G. G. et al. Chaotic Krill Herd algorithm. *Inf. Sci.* **274**, 17–34. 10.1016/j.ins.2014.02.123 (2014).

[21] Frazier, P.I. A Tutorial on Bayesian Optimization. 10.48550/arXiv.1807.02811 (2018). arXiv:1807.02811 [stat].

[22] Zhan, Z. H. et al. Adaptive particle swarm optimization. *IEEE Trans. Syst. Man Cybern. Part B (Cybern.)* **39**(6), 1362–1381. 10.1109/TSMCB.2009.2015956 (2009). 10.1109/TSMCB.2009.2015956 19362911

[23] He, X., Zhao, K. & Chu, X. AutoML: A Survey of the State-of-the-Art. 10.1016/j.knosys.2020.106622. arXiv:1908.00709 [cs] (2021).

[24] Barshooi, A. H. & Amirkhani, A. A novel data augmentation based on Gabor filter and convolutional deep learning for improving the classification of COVID-19 chest X-Ray images. *Biomed. Signal Process. Control* **72**, 103326. 10.1016/j.bspc.2021.103326 (2022). 34777557 10.1016/j.bspc.2021.103326 PMC8576144

[25] Namiranian, F. & Latif, A. A new approach for digital image segmentation with genetic algorithm and random forest. *Signal Data Process.* **20**(4), 35–44. 10.61186/jsdp.20.4.35 (2024).

[26] Xiao, X., Yan, M., Basodi, S. et al. Efficient hyperparameter optimization in deep learning using a variable length genetic algorithm. 10.48550/arXiv.2006.12703arXiv:2006.12703 [cs] (2020).

[27] Fatyanosa, T. N. & Aritsugi, M. An automatic convolutional neural network optimization using a diversity-guided genetic algorithm. *IEEE Access* **9**, 91410–91426. 10.1109/ACCESS.2021.3091729 (2021).

[28] Li, C., Jiang, J., Zhao, Y., et al. Genetic Algorithm based hyper-parameters optimization for transfer Convolutional Neural Network. 10.48550/arXiv.2103.03875arXiv:2103.03875 [cs] (2021).

[29] Zhang, Y. et al. A convolutional neural network based on an evolutionary algorithm and its application. *Inf. Sci.* **670**, 120644. 10.1016/j.ins.2024.120644 (2024).

[30] Raiaan, M. A. K. et al. A systematic review of hyperparameter optimization techniques in convolutional neural networks. *Decision Anal. J.* **11**, 100470. 10.1016/j.dajour.2024.100470 (2024).

[31] Mohakud, R. & Dash, R. Designing a grey wolf optimization based hyper-parameter optimized convolutional neural network classifier for skin cancer detection. *J. King Saud Univ. Comput. Inf. Sci.* **34**(8, Part B), 6280–6291. 10.1016/j.jksuci.2021.05.012 (2022).

[32] Wang, W. et al. CNN-based hybrid optimization for anomaly detection of Rudder system. *IEEE Access* **9**, 121845–121858. 10.1109/ACCESS.2021.3109630 (2021).

[33] Akan, S., & Akan, T. Battle royale optimizer with a new movement strategy. In *Handbook of Nature-Inspired Optimization Algorithms: The State of the Art: Volume I: Solving Single Objective Bound-Constrained Real-Parameter Numerical Optimization Problems* (eds Mohamed, A. et al.) 265–279. 10.1007/978-3-031-07512-4_10 (Springer International Publishing, 2022).

[34] Bansal, S. et al. Multi-objective genetic algorithm based deep learning model for automated COVID-19 detection using medical image data. *J. Med. Biol. Eng.* **41**(5), 678–689. 10.1007/s40846-021-00653-9 (2021). 34483791 10.1007/s40846-021-00653-9 PMC8408308

[35] Saberironaghi, A., Ren, J. & El-Gindy, M. Defect detection methods for industrial products using deep learning techniques: A review. *Algorithms* **16**(2), 95. 10.3390/a16020095 (2023).

[36] Usamentiaga, R. et al. Automated surface defect detection in metals: A comparative review of object detection and semantic segmentation using deep learning. *IEEE Trans. Ind. Appl.* **58**(3), 4203–4213. 10.1109/TIA.2022.3151560 (2022).

[37] Najaran, M. H. T. A genetic programming-based convolutional deep learning algorithm for identifying COVID-19 cases via X-ray images. *Artif. Intell. Med.* **142**, 102571. 10.1016/j.artmed.2023.102571 (2023). 37316095 10.1016/j.artmed.2023.102571 PMC10182835

[38] Sheta, A., Braik, M.S. & Aljahdali, S. Genetic algorithms: A tool for image segmentation. In *2012 International Conference on Multimedia Computing and Systems*, 84–90. 10.1109/ICMCS.2012.6320144. https://ieeexplore.ieee.org/abstract/document/6320144 (2012).

[39] Zheng, Z.R., Liu, Z.C., Liu, X.Y., et al. Genetic algorithm-based image preprocessing for volume rendering optimization. In *2009 IEEE International Symposium on IT in Medicine & Education*, 389–393, 10.1109/ITIME.2009.5236394. https://ieeexplore.ieee.org/abstract/document/5236394 (2009).

[40] Khan, A. H. et al. A genetic algorithm based feature selection approach for microstructural image classification. *Exp. Tech.* **46**(2), 335–347. 10.1007/s40799-021-00470-4 (2022).

[41] Kumazawa, N. et al. GA-based parameter optimization of image processing for contamination inspection of nonwoven fabrics. In *IECON 2022 – 48th Annual Conference of the IEEE Industrial Electronics Society*, 1–6. 10.1109/IECON49645.2022.9968436. https://ieeexplore.ieee.org/document/9968436 (2022).

[42] Jung, Y. J., Han, S. H. & Choi, H. J. Explaining CNN and RNN using selective layer-wise relevance propagation. *IEEE Access* **9**, 18670–18681. 10.1109/ACCESS.2021.3051171 (2021).

[43] Wang, Z. J. et al. CNN explainer: Learning convolutional neural networks with interactive visualization. *IEEE Trans. Visual Comput. Graph.* **27**(2), 1396–1406. 10.1109/TVCG.2020.3030418 (2021). 10.1109/TVCG.2020.3030418 33048723

[44] Guo, T. et al. Simple convolutional neural network on image classification. In *2017 IEEE 2nd International Conference on Big Data Analysis (ICBDA)*, 721–724 10.1109/ICBDA.2017.8078730https://ieeexplore.ieee.org/abstract/document/8078730 (2017).

[45] Munsarif, M. et al. Improving convolutional neural network based on hyperparameter optimization using variable length genetic algorithm for English digit handwritten recognition. *Int. J. Adv. Intell. Inform.* **9**(1), 66–78. 10.26555/ijain.v9i1.881 (2023).

[46] Cabot, J. H. & Ross, E. G. Evaluating prediction model performance. *Surgery* **174**(3), 723–726. 10.1016/j.surg.2023.05.023 (2023). 37419761 10.1016/j.surg.2023.05.023 PMC10529246

[47] Obi, J. C. A comparative study of several classification metrics and their performances on data. *World J. Adv. Eng. Technol. Sci.* **8**(1), 308–314. 10.30574/wjaets.2023.8.1.0054 (2023).

[48] Ahmad, S. et al. Confusion matrix-based modularity induction into pretrained CNN. *Multimed. Tools Appl.* **81**(16), 23311–23337. 10.1007/s11042-022-12331-2 (2022).

[49] Heydarian, M., Doyle, T. E. & Samavi, R. MLCM: Multi-label confusion matrix. *IEEE Access* **10**, 19083–19095. 10.1109/ACCESS.2022.3151048 (2022).

[50] Chicco, D., Tötsch, N. & Jurman, G. The Matthews correlation coefficient (MCC) is more reliable than balanced accuracy, bookmaker informedness, and markedness in two-class confusion matrix evaluation. *BioData Mining* **14**(1), 13. 10.1186/s13040-021-00244-z (2021). 33541410 10.1186/s13040-021-00244-z PMC7863449

[51] Riehl, K., Neunteufel, M. & Hemberg, M. Hierarchical confusion matrix for classification performance evaluation. *J. R. Stat. Soc. Ser. C: Appl. Stat.* **72**(5), 1394–1412. 10.1093/jrsssc/qlad057 (2023).

[52] Salmon, B. P., Kleynhans, W., Schwegmann, C. P. et al. Proper comparison among methods using a confusion matrix. In *2015 IEEE International Geoscience and Remote Sensing Symposium (IGARSS)*, 3057–3060, 10.1109/IGARSS.2015.7326461. https://ieeexplore.ieee.org/abstract/document/7326461 (2015).

